# Household solid waste management practices and perceptions among residents in the East Coast of Malaysia

**DOI:** 10.1186/s12889-021-12274-7

**Published:** 2022-01-05

**Authors:** Widad Fadhullah, Nor Iffah Najwa Imran, Sharifah Norkhadijah Syed Ismail, Mohd Hafiidz Jaafar, Hasmah Abdullah

**Affiliations:** 1 Environmental and Occupational Health Program, School of Health Sciences, Health Campus, Universiti Sains Malaysia, 16150 Kubang Kerian, Kelantan Malaysia; 2 School of Industrial Technology, Universiti Sains Malaysia, USM, 11800 Penang, Malaysia; 3 Department of Environmental and Occupational Health, Faculty of Medicine and Health Sciences, Universiti Putra Malaysia, 43400 Serdang, Selangor Malaysia; 4 Biomedicine Program, School of Health Sciences, Health Campus, Universiti Sains Malaysia, 16150 Kubang Kerian, Kelantan Malaysia

**Keywords:** Households’ Practices and Perception, Waste Segregation and Separation, Principal Component Analysis, Public Health, Solid Waste

## Abstract

**Background:**

Poor waste disposal practices hamper the progress towards an integrated solid waste management in households. Knowledge of current practices and perception of household solid waste management is necessary for accurate decision making in the move towards a more sustainable approach. This study investigates the household waste practices and perceptions about waste management in Panji, one of the sub-districts in Kota Bharu, Kelantan, Malaysia.

**Methods:**

A stratified random sampling technique using a cross-sectional survey questionnaire was used to collect data. A total of 338 households were interviewed in the survey and data were analyzed using SPSS. Chi-square goodness of fit test was used to determine the relationships between categorical variables, whereas Chi-square bivariate correlation test was performed to observe the correlation between the perceptions of waste segregation with socio-demographic background of the respondents. The correlation between perception of respondents with the locality, house type and waste type were also conducted. Principal component analysis was used to identify grouping of variables and to establish which factors were interrelated in any given construct.

**Results:**

The results of the study revealed that 74.3 % of households disposed of food debris as waste and 18.3% disposed of plastic materials as waste. The study also showed that 50.3% of the households segregate their waste while 49.7% did not. About 95.9% of the respondents were aware that improper waste management leads to disease; such as diarrhea and malaria. There were associations between locality, age and house type with waste segregation practices among respondents (Chi-square test, p<0.05). Associations were also found between locality with the perception of improper waste management which lead to disease (Chi-square test, p<0.05). Principal Component Analysis showed that 17.94% of the variance has high positive loading (positive relationship) with age, marital status and, type of house.

**Conclusion:**

This study highlights the importance to design waste separation programs that suit the needs of targeted population as a boost towards sustainable solid waste management practices.

**Supplementary Information:**

The online version contains supplementary material available at 10.1186/s12889-021-12274-7.

## Background

**Fig. 1:**
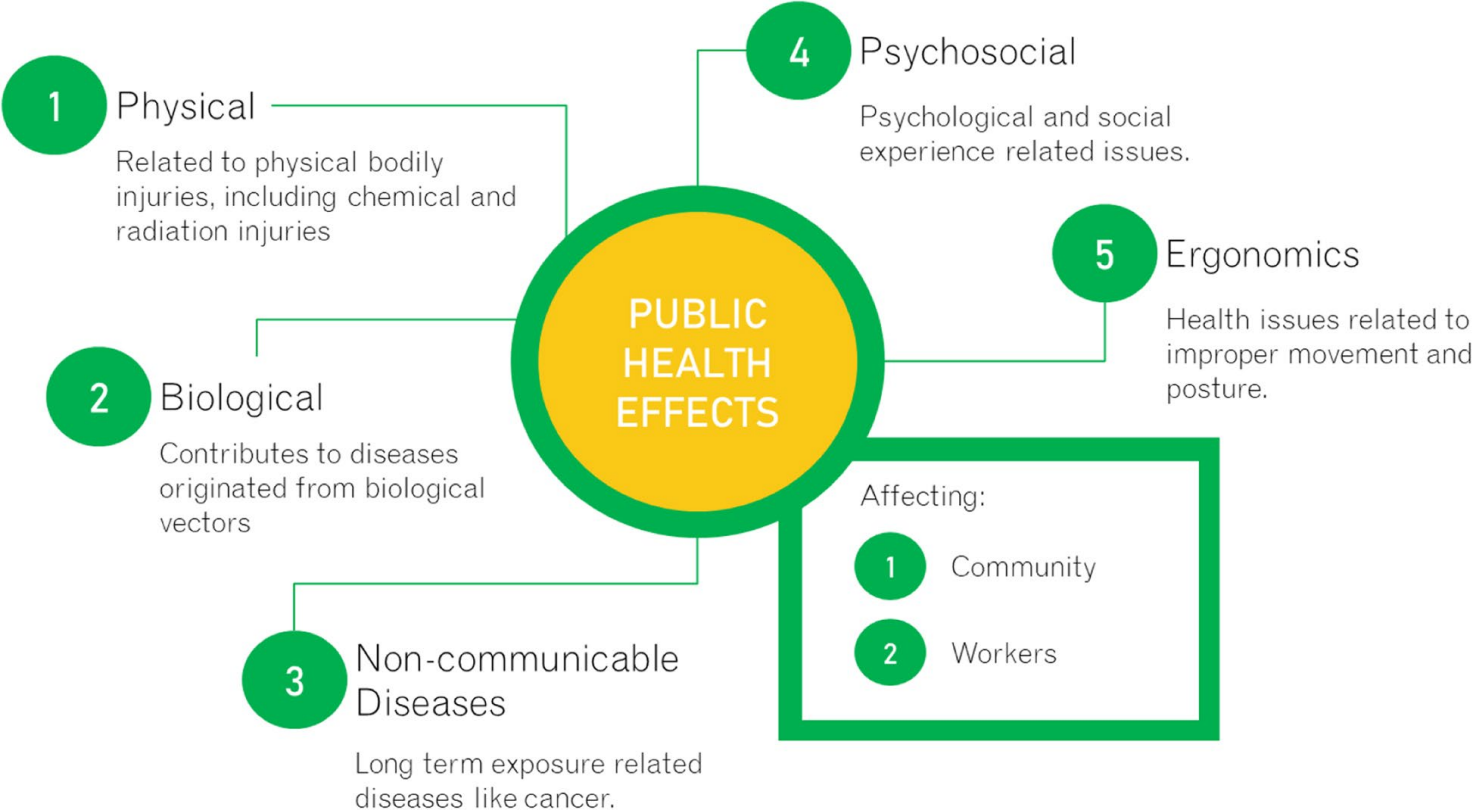
Effect of ineffective household solid waste management on public health

Solid waste management (SWM) in the majority of developing countries including Malaysia is dominated by open dumping due to lower capital, operational and maintenance cost in comparison with another disposal method [47]. This non-sanitary and non-engineered approach are without appropriate liners, gas collection and leachate collection and treatment, thereby exposing the surrounding environment with multiple air, water and soil pollution issues [15, 23]. The effects of the ineffective management of household solid waste on public health (Fig. 1) can be separated into physical, biological, non-communicable diseases, psychosocial and ergonomics health risks [6, 51, 77]. Contaminated soil, air and water provide breeding ground to biological vectors such as flies, rodents and insects pests. Many diseases are sequentially caused by these biological vectors, such as diarrhoea, dysentery, gastrointestinal problems, worm infection, food poisoning, dengue fever, cholera, leptospirosis and bacterial infection; irritation of the skin, nose and eyes; as well as respiratory symptoms [25, 41, 42, 52]. Exposure to gases generated by landfill waste such as methane, carbon dioxide, sulphur dioxide and nitrogen dioxide can produce inflammation and bronchoconstriction and can affect the immune cell. Hydrogen chloride and hydrogen fluoride released from the waste if deposited in the respiratory system, may cause cough, chest tightness and breathlessness [21].

Another category of health effects that can be closely related to household solid waste management is non-communicable diseases. Some studies estimated that the pollutions from the dumpsite might cause cancers (e.g. liver, pancreas, kidney, larynx) and non-Hodgkin lymphoma [8, 31, 51]. Other health effects under this category worth mentioning are birth defects, preterm babies, congenital disorders and Down’s syndrome [51, 52]. Apart from physical and biological effects, inefficient household waste management can lead to psychosocial effects such as disturbing odour, unsightly waste, and thinking, cognitive and stress-related problems [6, 51, 52, 74, 77]. Ergonomics is the final category of related health effects that is worth mentioning specifically for the working community of household waste management (Fig. 1). The risk of ergonomic issues is related to body posture, repetitive movement and excessive force movement [6].

Majority of the solid waste generated in Malaysia composed of organic waste with high moisture content [43], hence, the handling and waste separation at source is the most critical step in waste management [62]. The increasing amount of waste generated annually is also intensified by lack of land for disposing waste, questioning the sustainability of the current municipal solid waste (MSW) practices of using landfills [46]. Nevertheless, the lack of success in public participation to manage the solid waste is primarily rooted by the NIMBY (not in my backyard) attitude and the public perception that solid waste is a local municipal problem is highly prevalent among Malaysians [3]. Thus, most of the existing waste segregation practices by waste-pickers are mostly done in the informal sector as means of livelihood for the poor and additional source of income. On the other hand, this practice causes serious health problems, aggravating the socio-economic situation [10].

In Kelantan, the common practice of waste disposal in rural and remote areas is by burying and burning of waste (Kamaruddin et al. 2016) while in urban or semi-urban areas, stationary waste storage containers are provided mainly at the sides of the main road. Kota Bharu Municipal Council (KBMC) is the local authority responsible in providing stationary waste storage container at collection site of waste within Kota Bharu district, collecting the solid waste approximately 3 times a week by compactor vehicles and transporting waste to the dumpsite located in Beris Lalang, Bachok [27]. However, the flaws of SWM in Kelantan lies primarily in inadequate bin and waste collection provided by local authorities, KBMC mainly constrained by financial issues (Rahim et al 2012). House to house waste collection is also hard to be implemented owing to narrow lanes and alleys which are mostly inaccessible [61] due to the development practice and geographical area in the state. Therefore, the locals’ resort to burying and burning their wastes within their house compound which has always been the practice since decades ago.

Household waste is one of the primary sources of MSW comprising of food wastes, paper, plastic, rags, metal and glasses from residential areas. Household waste is among the solid wastes managed by KBMC in Kota Bharu covering 15 sub-districts including Panji. Panji has the highest population compared to the other sub-district; therefore, assessment of household SWM among the residents is important to address their awareness and practices for planning an effective form of SWM. Some of the key factors influencing the effectiveness of SWM is by considering the size of the family, their income [67], level of education [19] and the location of household [1]. This factor is also supported by Shigeru [66] that the characteristics of households determine their recycling behavior and that sociodemographic conditions vary across municipalities. Socio-economic status and housing characteristics also affect the amount of municipal waste and how they manage it [20]. Therefore, it is crucial to understand the characteristics and needs of various households in designing a suitable waste management program.

Efficient SWM system is now a global concern which requires a sustainable SWM primarily in the developing countries. This study is another effort in gearing towards sustainable waste management practices in Malaysia which is also in line with the United Nation Sustainable Development Goals encompassing SDG3 Good Health and Wellbeing and SDG 12 Responsible Consumption and Production. So far, limited studies were reported in the East Coast of Malaysia, particularly in Kelantan on waste management practices at the household level [61] which is highly required to improve the current practices including finding the prospect of whether proper at source-sorting in households is feasible to be implemented. This study provides a case study in Panji, Kota Bharu concerning the current household characteristics and awareness of managing household solid waste in Kelantan. The findings are crucial for the waste authorities in the process of designing and providing an effective and specific action plan in the area.

**Fig. 2:**
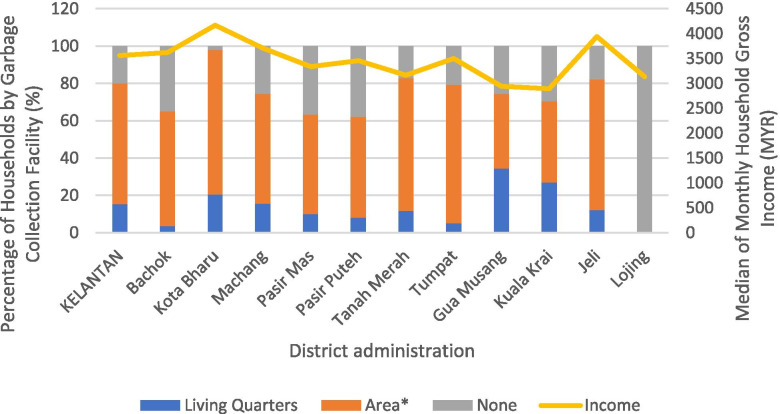
Percentage of households by garbage collection facilities and median monthly household income (MYR) for the districts in Kelantan

Figure 2 shows the percentage of households by garbage collection facilities and median monthly household income (MYR) for the districts in Kelantan. Kota Bharu is the district with the highest median monthly household gross income and percentage of garbage collection facilities. Apart from Lojing, which is located in the highlands, Bachok, Tumpat and Pasir Puteh are the districts with the lowest percentage of garbage collection facilities within 100m of the households. Meanwhile, Bachok (34.9%), Pasir Mas (36.6%), and Pasir Puteh (38%) households are without garbage collection facilities. The figure described the problem with household solid waste management in Kelantan. The major issues contributing to the problem are due to insufficient financial resources, lack of human labor, and transportation [61]. In one of the rural area in Kelantan, it was found that the solid waste management is considered inefficient due to a lack of knowledge in proper waste handling and the importance of segregating waste properly as proper waste handling start at home (Abas et al. 2020).

Household SWM is not a new issue, thus, published studies were found using survey and questionnaires and fieldwork studies. Waste characterization process was carried out by Kamaruddin et al. (2016) in 4 landfills in Kelantan. Nevertheless, they did not cover household waste knowledge, attitude and practices. Abdullah et al. [1] surveyed the household’s awareness on privatization of solid waste management and their satisfaction of the services offered but did not cover the health implications. Saat et al. [61] surveyed the practices and attitude on household waste management with a small sample size of less than 30 which limits its applicability to other region. Our study aimed to improve these previous studies by covering a wider sample size from the largest sub-district in Kelantan, Malaysia. The objective of this study is to assess the household SWM practices and perceptions among the residents of Panji vicinity in Kota Bharu district, Kelantan. Specifically, the objectives are to assess household SWM practices and perceptions in the Panji sub-district, to determine the association between socio-demographic characteristics or other factors and practices in SWM at the household level and to determine the association between socio-demographic characteristics or other factors and perceptions in SWM at household level.

## Methods

### Study area

**Fig. 3:**
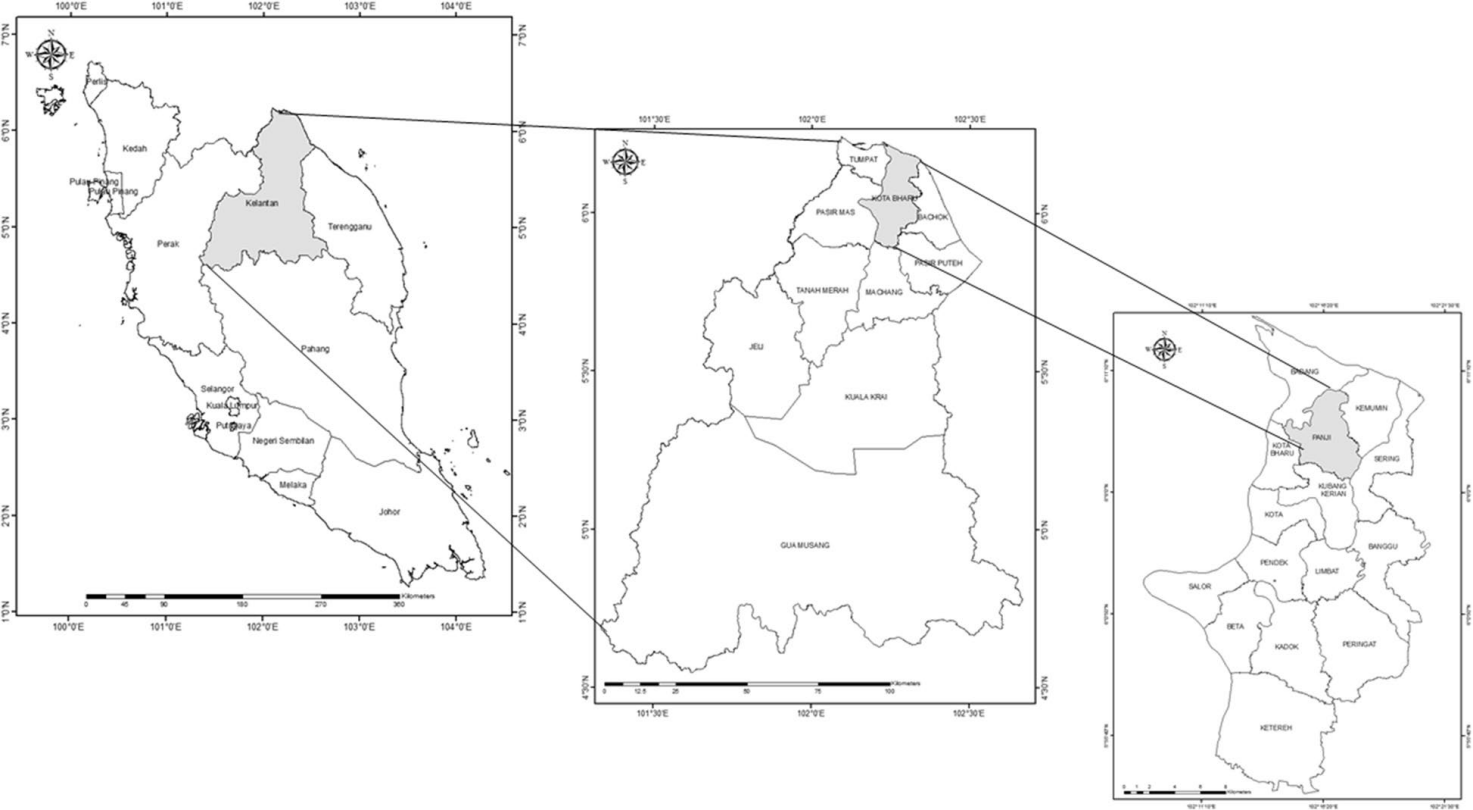
Location of the study area in Panji, Kota Bharu district, Kelantan, Malaysia (Source:ArcGis Software version 10.2; source of shape file: Department of Drainage and Irrigation, obtained with consent)

This study was conducted in Panji, Kota Bharu district, Kelantan, Malaysia (Fig. 3), located at the east cost of Peninsular Malaysia and has the highest population among the 15 sub-districts of Kota Bharu, the capital state of Kelantan. A total of 338 respondents were recruited in this study. The population of interest in this study involved residents in Kota Bharu district and considered only residents who have attained 18 years old and above. Sample unit is residents living in Kota Bharu district of more than a year and aged more than 18 years. The target population comprised all the households in Kota Bharu District (491,237); however, it is impossible to conduct a study with such a large number within a limited time period and inadequate financial budget. Therefore, a multi- stage random sampling technique was used in selecting the appropriate sample in order to evaluate the objectives of this study and to ensure that households in the districts had the same possibility of being included in the study (Dlamini et al., 2017). Initially, one district of Kelantan state (Kota Bharu) was selected out of 10 total districts. In the second stage, one sub-district of Kota Bharu District (Panji) was selected out of 15 total sub-districts. Eventually, 338 households were randomly selected as sample size. Convenient sampling was also used to select respondents due to time constraint and response obtained from target population. The localities involved were Kampung Tapang, Kampung Chempaka, Kampung Belukar, Kampung Panji, Taman Sri Iman, Taman Desa Kujid and Taman Bendahara.

### Data collection

A survey was conducted from January to May 2018. The questionnaire was translated from English to Malay language and the translation was done back to back and validated by experts in environmental science and public health field. A pilot test was conducted with a small sample size of ~30 to determine the suitability of the items in the questionnaire and the time taken by respondents to complete the questionnaires (Dlamini et al. 2017). Respondents were interviewed based on a questionnaire adopted and modified from Asante et al. [9]. The questionnaire involved two phases; the first one was to determine the socio-demographic of the respondents, including gender, age, types of housing, religion, educational level, occupation and the number of occupants in the household. Part two was an assessment to determine the status of household management of solid waste. The questionnaire included both open and closed questions (Dlamini et al. 2017). The closed questions were designed for ease of answering by the respondents with the aim of collecting the maximum appropriate responses, whereas the open questions are intended to encourage respondents to provide further elaboration on certain questions. The reliability of Cronbach’s alpha test of this questionnaire was found to be acceptable (α=0.71). Ethical approval for this study was obtained from the Ethic Committee of Universiti Sains Malaysia (USM/JEPeM/17100560).

### Data analysis

Data were analyzed using IBM Statistical Package for Social Science (SPSS) version 24.0. Descriptive analyses were used to report the frequency and percentage of socio-demographic patterns, method of household waste disposal and perceptions of household toward waste management. Chi-square goodness of fit test was used to determine the relationships between categorical variables, which allow us to test whether the observed proportions for a categorical variable differ from the hypothesized proportions [24]. The null hypothesis of the Chi-Square test is that no relationship exists on the categorical variables in the population; they are independent. Chi-square bivariate correlation test was performed to observe the correlation between the perceptions of waste segregation with socio-demographic background of the respondents [29]. The correlation between perception of respondents with the locality, house type and waste type were also conducted. Principal component analysis (PCA) was conducted to identify grouping of variables and to establish which factors were interrelated in any given construct, where a set of highly inter-correlated measured variables were grouped into distinct factors [24]. The Kaiser-Meyer-Olkim (KMO) Measure of Sampling Adequacy and Bartlett's Test of Sphericity was performed to evaluate the data's suitability for exploratory factor analysis [69].

## Results

### Socio-demographic Characteristics and Respondents Background in Panji sub-district

We first report descriptive statistics for all variables before discussing results from correlation analysis of socio-demographic factors and respondent’s background with household solid waste management (SWM) practices and perceptions. We then present the Principal Component Analysis (PCA). Table 1 represents the socio-demographic background and characteristics of the respondents in this study. Most of the respondents are from Kg. Belukar (N=125, 37%), followed by Kg. Panji (N=61, 18%), the rest are from Kg. Tapang (N=33), Kg. Chempaka, Taman Desa Kujid, Taman Sri Iman (N=30, respectively) and from Taman Bendahara (N=29). Majority of the respondents are female (N=182, 53.8%) and age between 35 to 49 years old (N=91, 26.9%). Most of the respondents have completed secondary education (N=194, 57.4%) and 31.1% have completed their degree or diploma (N=105). Majority of the respondents are married (75.7%), Muslim (97%) and earned between MYR 1000 to 2000 per month. About 32% of the respondents are self-employed and lived in a bungalow house type (30.5%). Most of the household consist of 4 to 6 occupants (53.6%). Majority of them cook at home (91.4%) on daily basis (68.6%). The Chi-square test shows that there is a significant difference among all categorical variables except for gender (χ ^2^= 2.000, p = 0.157).

**Table 1:** Socio-Demographic Characteristics and of Respondent’s Background in Panji sub-district (N = 338)

Variable	Description	Frequency (N)	Percentage (%)	Chi-square (p-value)
Locality	Kg. Chempaka	30	8.9	158.54 (<0.001)
	Kg Belukar	125	37.0	
	Kg Panji	61	18.0	
	Kg Tapang	33	9.8	
	Taman Desa Kujid	30	8.9	
	Taman Sri Iman	30	8.9	
	Taman Bendahara	29	8.6	
Gender	Male	156	46.2	2.00 (0.157)
	Female	182	53.8	
Age	18-24	46	13.6	59.81 (< 0.001)
	25-29	50	14.8	
	30-34	59	17.5	
	35-49	91	26.9	
	50-65	76	22.5	
	>65	16	4.7	
Level of Education	Primary	27	8.0	394.16 (< 0.001)
	Secondary	194	57.4	
	Tertiary (Diploma / Degree)	105	31.1	
	Professional (Master / Phd)	10	3.0	
	Missing	2	0.6	
Marital status	Single	77	22.8	296.53 (< 0.001)
	Married	256	75.7	
	Divorced	5	1.5	
Religion	Muslim	328	97.0	617.62 (< 0.001)
	Buddha	9	2.7	
	Christian	1	.3	
Monthly income	<1k	80	23.7	159.72 (< 0.001)
	1-2k	111	32.8	
	2-3k	84	24.9	
	4-5k	44	13.0	
	5-10k	18	5.3	
	Missing	1	0.3	
Occupation	Self employed	108	32.0	170.02 (< 0.001)
	Private sector	58	17.2	
	Housewife	66	19.5	
	Civil servant	67	19.8	
	Retiree	23	6.8	
	Student	8	2.4	
	Others	8	2.4	
Residential house type	Bungalow	103	30.5	52.50 (< 0.001)
	Semi detached	37	10.9	
	Terrace	50	14.8	
	Village	52	15.4	
	Others	96	28.4	
Number of occupants living in the household	1-3	98	29.0	68.92 (< 0.001)
	4-6	181	53.6	
	>6	59	17.5	
Cook at home	No	29	8.6	231.95 (< 0.001)
	Yes	309	91.4	
Cooking frequency	Not cooking	29	8.6	513.10 (< 0.001)
	Daily	232	68.6	
	2 times a week	23	6.8	
	3 times a week	48	14.2	
	Once a week	6	1.8	

### Proportion of Household Solid Waste Disposed by respondents in Panji Sub-District

**Fig. 4:**
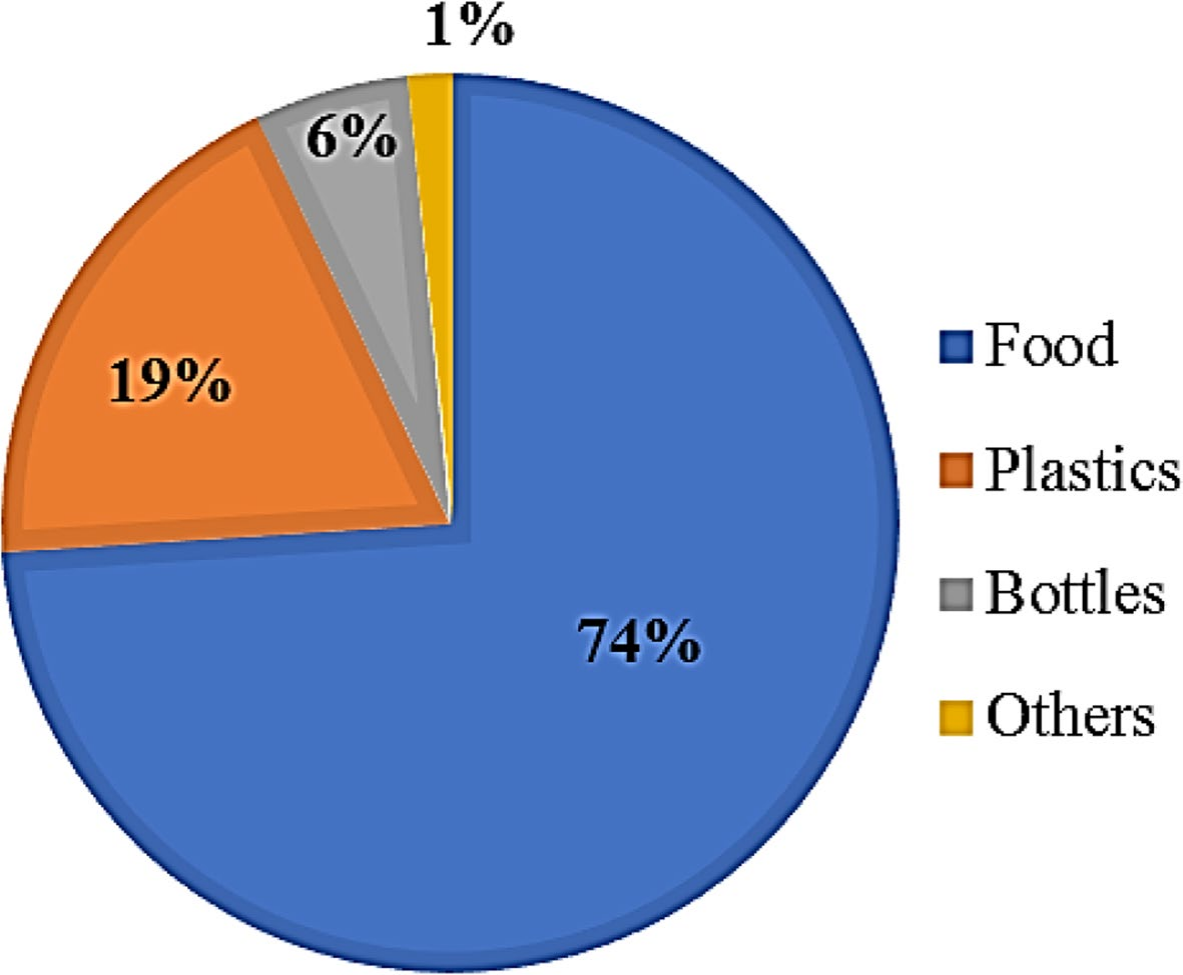
Types of waste disposed by household in Panji district

Figure 4 represents the type of waste disposed of by respondents in the study. More than half (74.38%) of the waste disposed by household is food debris, followed by plastic waste (19.01%) and bottles (5.79%) while the rest accounts for 0.83%.

### Household SWM practices and perceptions among respondents in Panji sub-district

Table 2 shows the household waste management practices and perceptions among respondents in Panji district. In terms of the household SWM practices, about 170 of the respondents (50.3%) segregate their waste at home while the remaining 168 respondents (49.7%) did not practice waste segregation at home. There is no significant difference between those who segregate waste at home and those who don’t (χ^2^=0.12, p=0.91). As shown in Fig. 1 and Table 2, the major type of waste disposed by respondents are food (N=251, 74.3%). A significant difference was found among the different type of waste disposed (χ^2^=656.56, p<0.001). Out of the 338 respondents interviewed, 75.4% of the respondent themselves normally carries their household waste to the allocated bin or waste collection point provided by the local authority. Majority of the respondents (323 respondents) agree that the waste disposal site provided by the local authorities were appropriate (95.6%) relative to 15 respondents who disagree (4.4%). A significant difference was found between those who responded that appropriate waste disposal site was provided and those who do not (χ2=280.66, p<0.001).

**Table 2:** Household SWM practices and perceptions among respondents (N = 338).

Variable	Description	Frequency	Percentage	Pearson chi-square (p-value)
Respondents’ practice waste segregation at home	Yes	170	50.3	0.12 (0.91)
	No	168	49.7	
Respondents’ practices: Type of waste disposed by respondents	Food	251	74.3	656.56 (< 0.001)
	Bottles	19	5.6	
	Plastic	62	18.3	
	Others	5	1.5	
	Missing	1	0.3	
Respondents’ practices: Who normally carries the household waste to the allocated bin provided by the local authority	Own self	255	75.4	460.75 (< 0.001)
	Children	17	5.0	
	Paid collector	32	9.5	
	Others	34	10.1	
Respondents’ perceptions that appropriate waste disposal site is provided by the local authority	Yes	323	95.6	280.66 (< 0.001)
	No	15	4.4	
Respondents’ perceptions on the importance of proper waste management	Important	337	99.7	-
	Missing	1	0.3	
Respondents’ perceptions toward who is responsible to clean the residential area	The residence	211	62.4	441.85 (< 0.001)
	Community	38	11.2	
	District council	82	24.3	
	Private waste operator	5	1.5	
	Missing	2	.6	
Respondents’ perceptions on improper waste management contribute to disease occurrence	Yes	324	95.9	594.68 (< 0.001)
	No	9	2.7	
	Not sure	5	1.5	
Respondents’ perceptions of disease that may relate to improper waste management	Malaria	74	21.9	188.24 (< 0.001)
	Typhoid	21	6.2	
	Diarrhea	103	30.5	
	Others	138	40.8	
	Missing	2	.6	
Respondents’ perceptions: How many respondents have knowledge/awareness about proper waste disposal?	Yes	314	92.9	248.82 (< 0.001)
	No	24	7.1	
Respondents’ perceptions: Element that motivates the household occupants to dispose waste properly	Cleanliness	275	81.4	352.80 (< 0.001)
	Fear of illness	42	12.4	
	Odor	21	6.2	

Most of them also have the perception that proper waste management is important (99.7%). More than half (62.4%) of the respondent agrees that it is their responsibility to clean the waste in their residential area while 24.3% suggested that it is the responsibility of the district council. Another 3.3% suggested it is the responsibility of the community members followed by private waste operators (1.5%). The majority (95.9%) of the respondents suggested poor waste management can contribute to disease occurrence, whereas 2.7% suggested it does not cause diseases and another 1.5% were unsure if it causes any diseases.

In terms of the household SWM perceptions, 40.8% of the respondents have responded that other diseases than diarrhea, malaria and typhoid are related to improper waste management. This is followed by diarrhea (30.5%) and malaria (21.9%). Majority of the participants responded that they have awareness on proper waste management (92.9%) and 81.4% responded that cleanliness is the main factor which motivates them to dispose the waste properly. The chi-square test shows that all variables under respondents’ perception differ significantly from the hypothesized values (Table 2).

### Relationship between socio-demographic characteristics, respondent’s background and household SWM practices (waste segregation practices)

Chi square analysis was performed to find out what factors contribute to waste segregation practices among the respondents (Table 3). Results indicate that waste segregation practice was correlated with the locality (χ^2^ = 43.35, p<0.001). For instance, out of 29 respondents in Taman Bendahara, all of them segregate their waste (100%). This trend was also observed for Taman Desa Kujid where most of the respondents segregate their waste (22 out of 30, 73.3%). In contrast, most of respondents from the village, did not segregate their waste. For example, out of 125 total number of respondents in Kg Belukar, 53 of them segregates their waste (42.4%) while 72 of them did not (57.6%).

**Table 3:** Correlation between respondents’ socio-demographic characteristics and practices in solid waste management at household level (Practice waste segregation)

Variables		Practice waste segregation				Pearson chi-square (p-value)
		Yes ^(N = 17)^		No ^(N = 16)^		
		Frequency	Row %	Frequency	Row %	
Locality	Kg. Chempaka	15	50.0%	15	50.0%	43.35 (<0.001)*
	Taman Desa Kujid	22	73.3%	8	26.7%	
	Taman Sri Iman	11	36.7%	19	63.3%	
	Kg Belukar	53	42.4%	72	57.6%	
	Kg Panji	24	39.3%	37	60.7%	
	Kg Tapang	16	48.5%	17	51.5%	
	Taman Bendahara	29	100.0%	0	.0%	
Gender	Male	82	52.6%	74	47.4%	.596 (0.440)
	Female	88	48.4%	94	51.6%	
Age	18-24	31	67.4%	15	32.6%	11.62 (<0.001)*
	25-29	19	38.0%	31	62.0%	
	30-34	30	50.8%	29	49.2%	
	35-49	39	42.9%	52	57.1%	
	50-65	43	56.6%	33	43.4%	
	>65	8	50.0%	8	50.0%	
Education level	Primary	13	48.1%	14	51.9%	6.188 (0.19)
	Secondary	90	46.4%	104	53.6%	
	Tertiary	61	58.1%	44	41.9%	
	Professional	6	60.0%	4	40.0%	
	Missing	0		2		
Marital status	Single	44	57.1%	33	42.9%	1.87 (0.17)
	Married	123	48.0%	133	52.0%	
	Divorce	3	60.0%	2	40.0%	
Monthly income	<1k	44	55.0%	36	45.0%	4.55 (0.47)
	1-2k	53	47.7%	58	52.3%	
	2-3k	40	47.6%	44	52.4%	
	4-5k	26	59.1%	18	40.9%	
	5-10k	7	38.9%	11	61.1%	
	Missing	0		1		
Occupation	Self employed	47	43.5%	61	56.5%	4.46 (0.62)
	Private sector	31	53.4%	27	46.6%	
	Housewife	32	48.5%	34	51.5%	
	Civil servant	37	55.2%	30	44.8%	
	Retiree	14	60.9%	9	39.1%	
	Student	5	62.5%	3	37.5%	
	Others	4	50.0%	4	50.0%	
Type of house	Bungalow	58	56.3%	45	43.7%	12.73 (0.03)*
	Semi detached	24	64.9%	13	35.1%	
	Terrace	20	40.0%	30	60.0%	
	Village	22	42.3%	30	57.7%	
	Other	46	47.9%	50	52.1%	
Number of occupants per household	1-3	54	55.1%	44	44.9%	2.366 (0.31)
	4-6	84	46.4%	97	53.6%	
	>6	32	54.2%	27	45.8%	
Cooking at home	Yes	152	49.2%	157	50.8%	1.75 (0.19)
	No	18	62.1%	11	37.9%	

A significant correlation was found between waste segregation practice and age (χ^2^ =11.62, p<0.001). Based on the age range of the total number of respondents, respondents at the age of 50-65 years old are those who segregated more than the rest (N=43) and those at the age of 35-49 are those who did not segregate their waste the most (N=52 in Table 3). The type of house was significantly correlated with waste segregation practice (χ^2^ =12.73, p=0.03). The respondents who live in bungalow houses are those who segregate the most (N=58). Those who live in semi-detached houses also have more respondents (N=24) segregating their waste than those who did not (N=13). Meanwhile those who live in other type of houses, terrace, village and others have more respondents who did not segregate their waste (Table 3). Other variables, gender, education level, marital status, monthly income, occupation, the number of persons per household and the practice of cooking at home did not show any significant correlation with waste segregation practice (p>0.05, Table 3).

### Relationship between respondent’s background and household SWM practices (the type of waste disposed) from the household in Panji sub-district

The chi-square test was also conducted to determine the relationship between socio-demographic characteristics, respondent’s background and the type of waste disposed. There is a significant correlation between locality with the waste type disposed in Panji district (Table 4). All localities showed that food waste was the major type of waste being disposed of from the households. A significant correlation was also found between respondents living in different house types with type of waste disposed. Most of the respondents who live in bungalows (N = 81) and other type of house (N = 78) disposed of food as the main waste from their households. Other characteristics were not significantly correlated with type of waste.

**Table 4:** Correlation between socio-demographic characteristics, respondent’s background and type of waste disposed from the household at Panji district

		Waste type				X^2^ (p-value)
		Food	Bottles	Plastic	others	
Locality	Kg. Chempaka	18	6	6	0	43.67 (0.002)*
	Taman Desa Kujid	25	0	5	0	
	Taman Sri Iman	21	1	7	0	
	Kg Belukar	83	7	30	5	
	Kg Panji	49	4	8	0	
	Kg Tapang	29	0	4	0	
	Taman Bendahara	26	0	3	0	
Gender	Male	119	9	28	0	5.338 (0.254)
	Female	132	10	34	5	
Age	18-24	36	3	7	0	23.67 (0.26)
	25-29	36	4	8	1	
	30-34	47	0	11	1	
	35-49	71	5	12	3	
	50-65	48	7	21	0	
	>65	13	0	3	0	
Marital status	single	55	8	14	0	9.28 (0.32)
	married	192	10	48	5	
	divorce	4	1	0	0	
Number of persons per household Education level	1-3	77	6	15	0	5.930 (0.655)
	4-6	129	11	37	3	
	>6	45	2	10	2	
	Primary	21	1	5	0	11.896 (.751)
	secondary	135	12	43	4	
	tertiary	85	6	12	1	
	Professional	9	0	1	0	
Type of house	Bungalow	81	7	12	2	49.745 (<0.001)
	Semi detached	29	3	5	0	
	Terrace	33	1	15	1	
	Village	30	7	13	2	
	Other	78	1	17	0	
Cook at home	Yes	232	16	55	5	2.729 (0.604)
	No	19	3	7	0	

*p<0.05

### Correlation between respondents’ background (locality and/ or house type) and the perception in household SWM (appropriate site of household waste disposal provided by the local council and improper waste management contribute to disease occurrence)

Correlation analysis was also performed to determine what factors contribute towards the perception of household SWM in Panji district. No significant correlation was found between different locality with the appropriate waste disposal site provided (p = 0.152) as most of the locality has an appropriate disposal site (Table 5). There was also no significant relationship between type of house with appropriate disposal site provided by the local council (p=0.131). On the other hand, significant correlation was found between locality and the respondent’s perceptions on improper waste management which contribute to disease occurrence (p=0.042). Out of all localities, majority of the respondents from Kg Belukar has the perception that improper waste management contributes to disease occurrence (Table 5).

**Table 5:** Correlation between locality and/or house type and the perception in household SWM

Variables		Appropriate disposal site provided		Pearson Chi-square	
		Yes ^(N = 170^)	No ^(N = 168)^	(p-value)	
Locality	Kg. Chempaka	30	0	8.074 (0.152)	
	Taman Desa Kujid	30	0		
	Taman Sri Iman	30	0		
	Kg Belukar	115	10		
	Kg Panji	57	4		
	Kg Tapang	32	1		
	Taman Bendahara	29	0		
Type of house	Bungalow	96	7	8.486 (0.131)	
	Semi detached	37	0		
	Terrace	49	1		
	Village	47	5		
	Other	94	2		
Locality	Kg. Chempaka	Improper waste management contribute to disease occurrence			18.887 (0.042)*
		Yes	No	Not sure	
		29	1	0	
	Taman Desa Kujid	29	1	0	
	Taman Sri Iman	29	1	0	
	Kg Belukar	119	2	4	
	Kg Panji	60	0	1	
	Kg Tapang	29	4	0	
	Taman Bendahara	29	0	0	

*p<0.05

### Principal component analysis (PCA)

Principal Component Analysis (PCA) is a dimension-reduction tool that can be used to reduce a large set of variables to a small set that still contains most of the information in the original large set [24]. It converts a set of observations of possibly correlated variables (entities each of which takes on various numerical values) into a set of values of linearly uncorrelated variables called principal components [37]. This transformation is defined in such a way that the first principal component has the largest possible variance (that is, accounts for as much of the variability in the data as possible), and each succeeding component in turn has the highest variance possible under the constraint that it is orthogonal to the preceding components.

PCA in this study was performed to determine the variables that influence or related to waste segregation behavior among respondents. Table 6 highlight the PCA analysis to illustrate the component factors that influence waste segregation behavior among respondents in this study. Only 13 significant variables were highlighted in the table with the factor loading of more than 0.5. Only factor loadings value >0.5 are considered for selection and interpretation due to having significant factor loadings influence the acceptable KMO value that represent a significant correlation for the PCA model in the study. The PCA generates four principal components that represent 48.26% of the total variance in the variables dataset and produced an acceptable KMO value of 0.603 (more than 0.5). Bartlett’s test of sphericity showed that PCA could be applied to the data at the p< 0.001 level. This approved that the data met the requirements for factor analysis [24, 69].

**Table 6:** The component matrix

	Principal Component (PC)			
	PC1	PC2	PC3	PC4
Age	.829			
Marital status	.707			
Locality	-.631			
Type of house	.569			
Education level	-.536			
Cooking at home		.597		
Cooking frequency		.698		
Improper waste management contribute to disease occurrence			.624	
Element of cleanliness motivating the household in waste disposal			.614	
Monthly income			-.562	
The respondent themselves brought the waste to the communal bin provided by the local council				.576
Number of persons living in a household				-.532
The residences are among those responsible party to clean the residential area				.525
% of variance	17.94	10.93	9.96	9.42

The component matrix produced in PCA showed that PC1 represents 17.94% of the variance with high positive loading (positive relationship) on age, marital status and, type of house (Table 6). This pattern indicates that age, married and type of house were the group that segregates their waste the most. This group of community can be proposed as the target to actively participate in waste management practices within the district. In contrast, locality and education have negative loading or negative relationship with the segregation activity. As a result, policy makers should increase educational activities on proper household waste practices and management related issues to minimize both the environmental and health impacts of household waste practices among the population.

PC2 represents 10.93% of the variance with high loadings on cooking at home and cooking frequency. This pattern implies that those who cook at home and frequently cook were among the most respondents who practice waste segregation. However, no consequences can be drawn about individual factors as these may have the opposite relationship to the observed factor in other components. Similar trend was observed for PC3 whereby 9.96% of the data variance has high loading on the perception of the respondents towards waste management. High loading was observed on perception that improper waste management contributes to disease occurrence and the cleanliness is the main element that motivates them to segregate. PC3 has high negative loading with monthly income. This result suggests that respondents with low income are those who segregate more.

Meanwhile, PC4 represents 9.42% of the data variance. Variables that have high positive loadings were the respondents who brought the waste to the communal bin themselves, indicating that this group of respondents are those who segregate more. High positive loading was also found on the perception that residents are among those responsible for cleaning the residential area. The number of persons living in a household has negative loading in PC4, indicating that the higher the number of people lives in the household, the lesser chances of them to segregate the waste.

Extraction Method: Principal Component Analysis.

a 4 components extracted.

b Only cases for which Practice of waste segregation = Yes are used in the analysis phase.

## Discussion

This study explores the behavioral perspective in view that the way people manage waste is associated with their attitude and perception. Individual perception is governed by their background and present situation, shaped by values, moods, socials circumstances and individual expectation (Kaoje et al 2017). The results of this study are discussed from three aspects: (1) characterization of household solid waste management practices and perceptions among respondents (2) correlation between socioeconomic and respondent’s background with waste segregation practices and (3) correlation between socioeconomic and respondent’s background with household waste management perceptions. One of the primary intentions of acquiring the respondent’s characteristics was to understand the correlation between level of involvement in household SWM practices and the characteristics of the respondents.

Food waste was found as the major type of waste disposed by the communities in Panji sub-district (Fig. 1 and Table 2). Food waste has high moisture content and causes smell, which subsequently attracts disease vectors, such as flies, mosquitoes and cockroaches, and the proliferation of rodents, such as rats and mice, which pose threats to public health [68, 75]. Majority of the respondents were found to cook at home (N=309, 91.4%) and cook on a daily basis (N=232, 68.6%; Table 1) which suggests that composting should be incorporated as one of the main approaches for proper waste management practices in the community. Individual compost bin should be provided in each household coupled with adequate training on simple compost technique can be organized within the locality as a stage by stage process. Alternatively, community scale composting can be proposed to focus solely on food waste management which is currently a growing practice among Malaysians [38, 56]. This approach is gaining attention because of their lower energy footprint, ease of operation, need for lesser resources, lower operation and maintenance costs which have higher chances of public acceptance [32]. Food waste is organic waste which can decomposed and degraded into organic matter [33], which in turn can be used by the public to fertilize their garden soil. Most importantly, the training should emphasize on the practicality and feasible option of composting which is otherwise seen as a time-consuming and burdensome process [33].

Composting is beneficial to the environment by reducing greenhouse gases emissions and improvement of soil quality when applied to land. Furthermore, it is also in line with the circular economy concept by closing the loop of the system [14]. On the other hand, there are issues pertaining to its quality such as the nutrient and trace metal content. So, sorting the waste at source play a crucial role in minimising these impurities and collection systems play a fundamental role in removing some pollutants from wastes, especially organic fraction of municipal solid wastes, and improving compost quality [13]. One way to overcome this is by accommodating the waste collection and composting facilities with easy and convenient measurement of these contents which may be accessible by the community. Community composting programs should incorporate not only the step-by-step procedure of how to do composting but at the same time introducing easy to use kit or techniques applicable to the public and community such as test strip to measure the nutrients and trace metal [11]. In addition, by adding composting accelerators, the nutritional quality of the compost can be overcome. This factor can be done by developing a manual for public use.

The case of local composting at homes reduces transportation and collection cost by decreasing the amount of domestic waste carried to centralized composting facilities [76]. At the same time, household waste contains impurities and are widely distributed which hinders the efficiency of centralized composting facilities in disposing them. Centralized composting facilities in Asia suffer from low compost quality and poor sales [32]. As a result, community composting system at a smaller scale is more convenient within this region.

Composting is linked to diseases such as Aspergillosis, Legionnaire’s disease, histoplasmosis, paronychia and tetanus. In the case of Aspergillosis and Legionnaire’s disease, it may cause higher potential risk in large scale composting facilities compared to the smaller scale composting at home due to massive handling and agitating process in the former [26, 59]. Histoplasmosis have been associated with chicken manure used in composting, however it is not able to survive in a well-done composting process [39]. Therefore, disease spread can be minimised by having local composting at homes and community composting system at a smaller scale than centralized composting facility. The most important thing in minimising disease spread would be the practise of wearing gloves and face mask during this composting activity.

In this study, there was not much difference between the respondents who separated their waste and who did not (Table 2), which implies there is room for increasing the practice of waste segregation. Waste segregation practice is lacking in developing countries, most prominently in Asia ( [15, 48]; Vassanadumrongdee and Kittipongvises 2018) and African continents (Dlamini et al. 2017; Yoada et al. 2014). Since respondents lack adequate knowledge on the critical importance of waste separation at source in general, the volume of municipal solid waste dumped in landfill sites are progressively increasing, thus jeopardizing the remaining landfill space at a faster rate than initially planned. Therefore, to alleviate this environmental problem in the developing countries in general and in Panji sub-districts, specifically, more focused and sustained public awareness programs, integrated with an enabling infrastructure, are required to change residents’ perceptions toward improved waste separation at source rates [49]. Additionally, the outcome of the waste segregation activities should be similarly emphasized and how waste minimization in the first instance, and waste segregation at source, will benefit and enhance the standard of living or life quality of households ([44]; Yoada et al. 2014 [49];).

The perceptions of the respondents towards waste management were generally good. About 99.7% reported that waste management is important, 62.4% report that it is the responsibility of them to manage waste (Table 2). Resident’s participation in waste management activities is one of the ways in maximizing the capture of source-segregated materials which can be facilitated by providing an associated infrastructure [58]. Nevertheless, there are still some respondents who felt that waste management is not their responsibility, but instead lies mainly on the district council, which highlights the general perception of some Malaysians that waste is a local municipal issue [46]. About 95.9% of the respondents were aware that improper waste management leads to sicknesses or diseases, which implies that most of the households were aware of the health implication of waste. The management of MSW in developing Asian countries is driven by a public health perspective: the collection and disposal of waste in order to avoid the spread of disease vectors from uncollected waste [5]. The perception of the remaining 2.7% that waste management does not cause disease and 1.5% who were unsure need to be changed by targeting this group as a follow up program focusing on waste management and health issues. The respondents also have adequate level of awareness and knowledge about proper waste management (92.9%). This high level of awareness is because of several reasons for properly disposing of waste, including cleanliness as the major factor (81.4%), followed by fear of illnesses (12.4%), and odor (6.2%).

Most of the respondents thought that improper waste management could lead to diarrhea and malaria (Table 2). Diarrhea and waste management is associated with environmental factors such as waste disposal mechanism. House-to-house waste collection has been shown to decrease the incidence of malaria compared to other waste collection method [7]. Hence, this implies the possibility of malaria incidence in areas which burn their waste and areas which are inaccessible by any waste collection. Other diseases could be related to typhoid, dysentery, cholera, respiratory infections and injury [42]. Proper waste management can lead to improvement in the quality of the environment and public health while, mismanagement of waste can be implicated with water, soil and air pollutions [1], breeding of mosquitos, which in turn, causes disease [15, 68]. Although knowledge and awareness are acceptable among the respondents, this perception did not inculcate into waste segregation practices. In order to bridge the gap between awareness and behavior change, it is necessary for individuals to understand the importance of their role in how to do it and why it is important to do so [34]. More focused, detailed and continuous awareness and knowledge should be emphasized on this aspect specifically in the topics of environmental cleanliness, drainage systems, the recycling process in theory and practice, and a proper way to dispose of wastes [61].

Our findings have reported that socio-demographic factors (age, marital status) and respondents’ background (locality and house types) have influenced the household waste practices and perceptions in Panji sub-district (Tables 3, 4, 5 and 6). Age is associated with the maturity of the person which plays a significant factor in impacting their level of awareness on environmental health and sanitation ([12, 17]; Meneses and [40, 45]). The result of our study is consistent with the findings by Fan et al. [22] that older individuals prefer to engage more in waste sorting activities than young people in Singapore.

On the other hand, the number of children in the household may be a significant factor that influence waste separation. This for instance has been mentioned in Xu et al., (2017), where the intention of middle-aged adults towards behaving a more eco-friendly system was affected by critical social reference groups around them, such as the interaction with family or the motivation, especially children, and/or the consideration of the health situation of the whole family.

However, in other studies such as in Ittiravivongs [28] and Vassanadumrongdee & Kittipongvises (2018), socio-demographic variables became insignificant factors that influenced waste segregation participation. Knussen et al., [36] & White & Hyde [73] also indicate that the strongest variable influence participation in waste segregation program was past behaviour on regular source separation at home or recycling habit. Having waste separation in the office also could have positive influence on source separation intention, which is consistent with the study of Saphores et al. [64].

Considering number of children in the analysis is beyond the scope of this paper. Our result indicates that there is no significant difference in the waste segregation practice by the number of occupants in the household (χ^2^= 2.36, p = 0.31). For instance, the results show 54.2% of household with more than 6 occupants practice waste segregation, as compared to those who are not at 45.8%. This would suggest that the number of children in the house could be less influence on the waste segregation practice or vice versa. Future study may consider number of children in the family as one of the variables to be tested to confirm the hypothesis.

It was interesting to note that the types of housing in the case study were found to contribute heavily to the practices and perceptions of household waste management. Respondents who lived in bungalows (30.5%) and other type of houses than semi-detached, terrace and village (28.4%) are most likely to segregate their waste. Bungalows are associated with high income areas in Malaysia [53], which could be related to waste collection services are provided from these areas and possibly these households subscribe to this service. Potentially, these types of houses also have more space to be allocated for waste sorting than the other type of houses.

Other socio-demographic characteristics such as gender, education level and monthly income did not influence the practices and perceptions of the respondents. There were no significant associations between gender and waste segregation practices (χ^2^=0.596, p=0.440). Our finding is contrasting to the study by Ehrampoush and Moghadam [18] which reported that gender is likely to have an influence on the perceptions of household SWM. This view is supported by Mukherji et al. [48] who found that women, because of traditional gender roles associated with their household activities, have a closer engagement with waste management at household level.

The level of education has been reported as an important factor that could influence people’s perception of household waste management [40]. In this study, most of the respondents received their education until secondary school (57.4%), followed by diploma or degree (31.1%) but this did not influence their household SWM practices and perception (χ^2^=6.188, p=0.19), in particular waste segregation practice (Table 3). The poor average income of respondents is considered a very important variable that could influence people’s perception and attitudes negatively on solid waste management system (Parfitt et al. 1994 [40];). But, this is not the case in our study as economic consideration appears not to play a major role in the respondent’s perception as well as attitude to solid waste management practices (χ^2^=4.55, p=0.47).

The outcome from the PCA analysis showed that age, marital status and type of housing are the factors which contributed the most to waste segregation practices at home. Our finding agrees with the study by Vassanadumrongdee and Kittipongvises (2018) which found that age and family with children have a positive influence on respondent's source separation. Age was also a determinant factor in waste management practices in other studies [2, 15]. With aging and married respondents, this could be highly related to the increasing sense of responsibility towards the environment and the importance of increasing the quality of life among household members. Types of housing could be related to either waste collection services were provided in these areas or that limited number of households subscribe to their service. Other studies in the literature have reported on the positive relationship between residence types and waste separation practices ([15]; Vassanadumrongdee and Kittipongvises 2018).

The high loadings on cooking at home and cooking frequency towards waste segregation practices indicate that these groups of respondents can be chosen for further interventions in terms of adopting proper waste management practices such as small-scale composting, recycling and waste minimization practices. The lifestyle of the respondents plays a significant role in the daily waste disposal practices in households (Yoada et al. 2014 [15];). The link between improper waste management practice and disease occurrence was also reported in studies in Ghana (Yoada et al. 2014 [2];). Their studies also reported that cleanliness was the main factor which motivates them to segregate the waste which is concurrent with the findings in this study.

Education is negatively related to waste segregation activity (Table 6), indicating that people with lower education are more willing to segregate their waste as compared to those with higher education. The likely reasons could be related to different lifestyle and time constraint to allocate purposely for waste sorting activities [15]. People with higher education level may be spending most of their time at the workplace, and not at home. However, more educational campaign should be promoted by emphasizing on the benefits of waste segregation activities. Sufficient knowledge, such as clear instructions provided in a communication and collection campaign, can increase the probability of waste separation behavior (Vassanadumrongdee and Kittipongvises S 2018).

The higher number of occupants living in the household is associated with a less likely chance of segregating the waste (Table 6). The result of our study is consistent with the study by Addo et al. [2] which reported that household sizes of 4 to 6 and above 7 were less likely to engage in the practice of waste management as compared to household size below 4 people. This is probably due to the household size tends to reduce the quantity of household waste and the practice of waste management. In contrast, studies by Osbjer et al. [54], indicate that waste management practice is associated with a higher number of people in the households, which could possibly be due to the need to handle waste generated by larger populations within the household.

One of the objectives of this study was to determine variables that influence waste segregation behavior among respondents. The PCA was adapted for this objective rather than correlation analysis for several reason. The correlation coefficient assumes a linear association where any linear transformation of variables will not affect the correlation. However, variables X and Y may also have a non-linear association, which could still yield a low correlation coefficient [30]. In addition, the correlation coefficient cannot be interpreted as causal.

It is possible that there is a causal effect of one variable on the other, but there may also be other possible explanations that the correlation coefficient does not take into account. Since several variables may influence respondent’s behavior on waste segregation activity at one time, the correlation coefficient analysis may not adequate to identify the significant variables and the connectivity between them accurately. Therefore, PCA was used to help us understand the connection between these variables as it can identify the correlation among the features efficiently.

There are thousands of features in the dataset that possible to highlight some trend or the influence of one factor to another. There are challenges to visualize the algorithm on all features efficiently especially when the performance of the algorithm may reduce with the bigger dataset. The PCA improve the algorithm performance by getting rid of correlated variables which don't contribute to the model and the analysis of the algorithms reduces significantly with less number of features. The Principal Components are also independent of one another. There is no correlation among them. It also reduces overfitting by reducing the number of features where it mainly occurs when there are too many variables in the dataset.

The scenario of the covid-19 pandemic contributes to a significant challenge in managing household waste management globally and specifically in developing countries. Waste management in the pandemic scenario requires consideration in SARS-CoV-2 transmission through MSW handling that includes survival time of the virus on the surfaces: population density and socioeconomic conditions [35]. In general, waste management phases (waste packing and delivering by the users; waste withdrawal; waste transport; and waste treatment) exposed the community and workers to direct contact with contaminated objects and surfaces; as well as contact with airborne droplets at a distance that may lead to the covid-19 [16]. Due to these reasons, waste management practices are designed to respond to the pandemic through changes in the collection system, allocation of treatment options, safety measure and priority separation, and functionality of circular economy strategies [72].

As a developing country, it is predicted that the effect of covid-19 on the waste management practices are more crucial due to the increase in disposable personal protective equipment at the household level and changes in eating habits, as a consequence of lifestyle disruptions and psychological stress due to lockdowns [4, 55]. Developing countries have a higher risk of waste and wastewater contamination, leading to significant public health issues [71]. Inefficient waste management practices such as insecure landfills, lack of technical knowledge, scientific and economic resources, and lack of waste emergency policies produce severe consequences to the community and workers [63, 65, 71].

In order to improve the level of household solid waste management in the study area and Malaysia in general, it is important to empower the key drivers. The key drivers can be categorized as institutional-administrative, technological, economical, and social drivers [70]. A strong policy that implements direct regulation and enforcement; provide economic incentives or disincentives; and inform, interact and engage with the community are required [60].

Household solid waste management technologies that are being practised globally are landfilling, incineration, pyrolysis, Refuse Derived Fuel (RDF), gasification, and anaerobic digestion [57]. As a developing country that focuses on solid waste management through landfilling, it is important to put extra attention on: i. decentralization of household solid waste management; ii. segregation at the source; iii. hygienic and safe handling; iv. flammable landfilll gasses handling; v. soil salinity from compost application; vi. Sustainable landfill management; vii. alternative markets for energy products; and viii. Implementation of the “pay as you throw” system [50].

### Practical Implications, Study Limitations and Future Perspectives

This study highlights that waste segregation practice among respondents are still low and food waste are mixed with other household waste. This study provides as a baseline data in the region where less study was emphasized.

Quantitative and qualitative approach were used in this study by adopting descriptive and statistical analysis to improve the significance of the issue. Despite the significance of some aspects of this study, further studies should be done to incorporate children and teenagers as the participants and a more detailed questionnaire incorporating detailed health implications. Apart from that, a cross-sectional survey using random sampling technique was used to assess the household SWM practices and perceptions among the residents. This study is also limited to only Panji sub-districts which requires a wider region to generalize the findings of the study. The survey questionnaires depend on self-reporting manner, which may be subject to bias. Further study is recommended to engage observation at houses or at the waste collecting points to complement the survey. Moreover, the association between household socio-economic factors and health implications were limited. Future study should address this factor for a more focused and sustained public awareness programs.

## Conclusions

The study found that the waste segregation practice among respondents can be considered as low, where the number of respondents who segregate their waste was equivalent to those who did not, which implies there is room for improvement. The main component of solid waste generated at home was largely food debris that has the potential to be composted and plastics that can be recycled, which were mainly disposed without separation. The local solid waste management authority should focus on utilizing this organic waste through a larger scale and wider involvement of the locals in composting program. The growth of small-scale community-based waste composting can act as a potential start up venue in accelerating this program, without the necessity of extensive investment by the local authority. The authority in the study area has provided appropriate waste disposal sites, but there are also some that were disposed in inappropriate sites. Majority of the respondents were also aware that improper waste management can lead to diseases. Age, marital status and, type of house was found to be the group that segregate their waste the most, indicating that respondents which fall under this category can be the target for further intervention programs. This study suggests the local authorities to design waste separation programs that suit the needs of targeted population, to ensure high participation rate among the community. Marketing and campaigns should emphasize the positive perception and attitude towards waste separation at home and also negative perception of non-participants. This study may provide authorities in Malaysia with baseline information to set the future implementations of waste segregation activities in households. This study also suggests focusing on inculcating community involvement in doing waste separation at source, waste reduction and recycling as a habit and way of life. The local authority may facilitate this activity by providing bins to segregate wastes, establishing waste banks and recycling facilities at a wider scale than the scattered existing ones. Both a top-down and bottom-up approach should work hand in-hand to realize the sustainable solid waste management as a success.

Nevertheless, acknowledging the limitations of the current study, a more detailed and thorough study should incorporate a wider region, in-depth association of waste separation programs and health implications. Combining survey questionnaire with statistical analysis act as a stepping stone to expand the study by engaging the community in actual waste separation activities. This can be done by initiating a collaboration between the local authority, the leader in a community and the residents itself as a pilot study. In addition, the findings of this study will serve as baseline evidence and pave the way for other researchers and policymakers to conduct more rigorous studies on this arena.

## Supplementary Information

**Additional file 1.**

**Additional file 2.**

## Data Availability

The datasets supporting the conclusions of this article are included within the supplementary material section.

## Abbreviations

SPSS: Statistical Package for Social Science
SWM: Solid Waste Management
MSW: municipal solid waste
NIMBY: not in my backyard
KBMC: Kota Bharu Municipal Council
SDG: Sustainable Development Goals
MYR: Malaysian Ringgit
PCA: Principal component analysis
KMO: Kaiser-Meyer-Olkim
RDF: Refuse Derived Fuel

## References

[1] Abdullah Z, Salleh MS, Ismail KNIK (2017). Survey of Household Solid Waste Management and Waste Minimization in Malaysia: Awareness, Issues and Practices. International Journal of Environmental & Agriculture Research (IJOEAR).

[2] Addo HO, Dun-Dery EJ, Afoakwa E, Elizabeth A, Ellen A, Rebecca M (2017). Correlates of domestic waste management and related health outcomes in Sunyani, Ghana: a protocol towards enhancing policy. BMC Public Health.

[3] Agamuthu P, Fauziah SH (2011). Challenges and issues in moving towards sustainable landfilling in a transitory country-Malaysia. Waste Manag Res.

[4] Aldaco R, Hoehn D, Laso J, Margallo M, Ruiz-Salmón J, Cristobal J, Kahhat R, Villanueva-Rey P, Bala A, Batlle-Bayer L (2020). Food waste management during the COVID-19 outbreak: a holistic climate, economic and nutritional approach. Sci Total Environ.

[5] Aleluia J, Ferrão P (2016). Characterization of urban waste management practices in developing Asian countries: A new analytical framework based on waste characteristics and urban dimension. Waste Manag.

[6] Aminuddin MSH, Rahman HA (2015). Health risk survey for domestic waste management agency workers: Case study on Kota Bharu Municipal Council (MPKB), Kelantan. Malaysia Int J Environ Sci Dev.

[7] Amoatey PK, Winter J, Kaemph C (2008) Solid Waste Disposal and the Incidences of Malaria: Any Correlation? Proceedings of the Second IASTED Africa Conference September 8-10, 2008 Gaborone, Botswana Water Resource Management (AfricaWRM 2008).

[8] Ancona C, Badaloni C, Mataloni F, Bolignano A, Bucci S, Cesaroni G, Sozzi R, Davoli M, Forastiere F (2015). Mortality and morbidity in a population exposed to multiple sources of air pollution: A retrospective cohort study using air dispersion models. Environ Res.

[9] Asante KP, Kinney P, Zandoh C, Vliet EV, Nettey E, Abokyi L, Owusu-Agyei S, Jack D (2016). Childhood Respiratory Morbidity and Cooking Practices Among Households in a Predominantly Rural Area of Ghana. Afr J Infect Dis.

[10] Aweng ER, Fatt CC (2014). Survey of Potential Health Risk of Rubbish Collectors from the Garbage Dump Sites in Kelantan, Malaysia. Asian J Appl Sci (ISSN: 2321 – 0893).

[11] Ayilara MS, Olanrewaju OS, Babalola OO, Odeyemi O (2020). Waste Management through Composting. Challenges Potent Sustain.

[12] Bradley CJ, Waliczek TM, Zajicek JM (1999). Relationship between environmental knowledge and environmental attitude of high school students. J Environ Educ.

[13] Cesaro A, Belgiorno V, Guida M (2015). Compost from organic solid waste: quality assessment and European regulations for its sustainable use. Resour Conserv Recycl.

[14] Chen T, Zhang S, Yuan Z (2020). Adoption of solid organic waste composting products: A critical review. J Clean Prod.

[15] Choon SW, Tan SH, Chong LL (2017). The perception of households about solid waste management issues in Malaysia. Environ Dev Sustain.

[16] Di Maria F, Beccaloni E, Bonadonna L, Cini C, Confalonieri E, La Rosa G, Milana MR, Testai E, Scaini F (2020). Minimization of spreading of SARS-CoV-2 via household waste produced by subjects affected by COVID-19 or in quarantine. Sci Total Environ.

[17] Eagles PFJ, Demare R (1999). Factors influencing children’s environmental attitudes. J Env Education.

[18] Ehrampoush MH, Mogahadam MB (2005). Survey of knowledge, attitude and practice of Yazd University of Medical Sciences students about solid wastes disposal and recycling. Iranian J Env Health Sci Eng.

[19] Ekere W, Mugisha J, Drake L (2009). Factors influencing waste separation and utilization among households in the Lake Victoria crescent. Uganda Waste Manag.

[20] Emery AD, Griffiths AJ, Williams KP (2003). An in-depth study of the effects of socio-economic conditions on household waste recycling practices. Waste Manag Res.

[21] EPQS (Expert Pannel on Air quality standards) (2009) Adendum to Guidelines for Halogens and Hydrogen Halides in Ambient Air. London; The stationary office.

[22] Fan B, Yang W, Shen X (2019). A comparison study of ‘motivation–intention–behavior’ model on household solid waste sorting in China and Singapore. J Clean Prod.

[23] Fauziah SH, Agamuthu P. Trends in sustainable landfilling in Malaysia, a developing country. Waste Manag Res. 2012:1–8. 10.1177/0734242X12437564 22455994

[24] Field A (2009) Discovering Statistics Using SPSS. 3rd Edition, Sage Publications Ltd., London.

[25] Gutberlet J, Uddin SMN (2017). Household waste and health risks affecting waste pickers and the environment in low-and middle-income countries. Int J Occup Environ Health.

[26] Huss A, Derks LAN, Heederik DJJ, Wouters IM (2020) Green waste compost as potential reservoirs of Legionella in the Netherlands. Clin Microbiol Infection 26 (2020) 1259.e1e1259.e3. 10.1016/j.cmi.2020.05.018 32470567

[27] Idris A, Inanc B, Hassan M (2004). Overview of waste disposal and landfills/dumps in Asian countries. J Mat Cycl Waste Manag.

[28] Ittiravivongs A (2011). Factors Influence Household Solid Waste Recycling Behaviour In Thailand: An Integrated Perspective. WIT Transactions on Ecology and the Environment. Volume 167, Pages 12. Paper 10.2495/ST110391.

[29] Ismail SNS, Zainal Abidin E, Hashim Z, Rasdi I, How V, Praveena SM, Karuppiah K, Ho YB, Ismail S, Mohamad S, Nik Azme NNA (2018). Disaster Debris Management during the 2014-2015 Malaysia Flood Incident. Mal J Med Health Sci.

[30] Janse RJ, Hoekstra T, Jager KJ, Zoccali C, Tripepi G, Dekker FW, et al. Conducting correlation analysis: important limitations and pitfalls. Clin Kidney J. 2021;1–6. 10.1093/ckj/sfab085. 10.1093/ckj/sfab085 PMC8572982 34754428

[31] Jarup L, Briggs D, de Hoogh C, Morris S, Hurt C, Lewin A, Maitland I, Richardson S, Wakefield J, Elliott P (2002). (2002) Cancer risks in populations living near landfill sites in Great Britain. Br J Cancer.

[32] Joshi P, Visvanathan C (2019). Sustainable management practices of food waste in Asia: Technological and policy drivers. J Environ Manag.

[33] Karim Ghani WAWA, Rusli IF, Biak DRA, Idris A (2013). An application of the theory of planned behaviour to study the influencing factors of participation in source separation of food waste. Waste Manag.

[34] Kirakozian A. Selective Sorting of Waste: A study of Individual Behaviours. GREDEG WP No. 2014:2013–49.

[35] Kulkarni BN, Anantharama V (2020). Repercussions of COVID-19 pandemic on municipal solid waste management: Challenges and opportunities. Sci Total Environ.

[36] Knussen C, Yule F, MacKenzie J, Wells M (2004). An analysis of intentions to recycle household waste: the roles of past behaviour, perceived habit, and perceived lack of facilities. J Environ Psychol.

[37] Li L, Ararel E, Jeuland M (2019). The drivers of household drinking water choices in Singapore: Evidence from multivariable regression analysis of perceptions and household characteristics. Sci Total Environ.

[38] Lim WJ, Chin NL, Yusof AY, Yahya A, Tee TP (2016). Food waste handling in Malaysia and comparison with other Asian countries. Int Food Res J.

[39] Londoño LFG, Leoń LCP, Ochoa JGM, Rodriguez AZ, Jaramillo CAP, Ruiz JMA, Taylor ML, Arteaga MA, Alzate MdPJ (2019) Capacity of Histoplasma capsulatum to Survive the Composting Process. Appl Environ Soil Sci. Volume 2019, Article ID 5038153, 9 pages 10.1155/2019/5038153.

[40] Longe EO, Longe OO, Ukpebor EF (2009). People’s Perception On Household Solid Waste Management in Ojo Local Government Area in Nigeria. Iran J Environ Health Sci Eng.

[41] Maheshwari R, Gupta S, Das K (2015) Impact of Landfill Waste on Health: An Overview. IOSR Journal of Environmental Science, Toxicol Food Technol 1(4): 17-23. e-ISSN: 2319-2402, p- ISSN: 2319-2399.

[42] Mamady K. Factors Influencing Attitude, Safety Behavior, and Knowledge regarding Household Waste Management in Guinea: A Cross-Sectional Study. J Environ Public Health. 2016:1–9. 10.1155/2016/9305768 PMC4820610 27092183

[43] Manaf LA, MAA S, NIM Z (2009). Municipal solid waste management in Malaysia: Practices and challenges. Waste Manag.

[44] Matter A, Dietschi M, Zurbrügg C (2013). Improving the informal recycling sector through segregation of waste in the Household- The case of Dhaka Bangladesh. Habitat International.

[45] Meneses G.D, Palacio AB (2005) Recycling behavior: A multidimensional approach. Environ Behav 37: 837–860.

[46] Moh YCA, Manaf L (2017). Solid waste management transformation and future challenges of source separation and recycling practice in Malaysia. Resour Conserv Recycl.

[47] Moh YC, Manaf LA (2014). Overview of household solid waste recycling policy status and challenges in Malaysia. Resourc, Convers Recycl.

[48] Mukherji SB, Sekiyama M, Mino T, Chaturvedi B (2016). Resident Knowledge and Willingness to Engage in Waste Management in Delhi. India Sustain.

[49] Mwanza BP, Mbohwa C, Telukdarie A (2018). Levers Influencing Sustainable Waste Recovery at Household Level: A Review. Procedia Manufact.

[50] Nanda S, Berruti F (2021). Municipal solid waste management and landfilling technologies: a review. Environ Chem Lett.

[51] Ncube F, Ncube EJ, Voyi K (2017). A systematic critical review of epidemiological studies on public health concerns of municipal solid waste handling. Perspect Public Health.

[52] Norsa’adah B, Salinah O, Naing NN, Sarimah A (2020). Community health survey of residents living near a solid waste open dumpsite in Sabak, Kelantan, Malaysia. Int J Environ Res Public Health.

[53] Omran AL, Mahmood A, Abdul Aziz H, Robinson GM (2009). Investigating Households Attitude Toward Recycling of Solid Waste in Malaysia: A Case Study. Int J Environ Res.

[54] Osbjer K, Boqvist S, Sokerya S, Kannarath C, San S, Davun H, Magnusson U (2015). Household practices related to disease transmission between animals and humans in rural Cambodia. BMC Public Health.

[55] Oyedotun TDT, Kasim OF, Famewo A, Oyedotun TD, Moonsammy S, Ally N, Renn-Moonsammy D-M (2020). Municipal waste management in the era of COVID-19: perceptions, practices, and potentials for research in developing countries. Res Glob.

[56] Petaling Jaya Municipal Council (MBPJ) (2010) Composting closing the loop at home. A household home composting program in Petaling Jaya Municipal Council. http://www.ecoideal.com.my/danidaurban/swmc/download/SWMC_CI_Composting%20at%20MBPJ.pdf.

[57] Potdar A, Singh A, Unnnikrishnan S, Naik N, Naik M, Nimkar I (2016). Innovation in solid waste management through Clean Development Mechanism in India and other countries. Process Saf Environ Prot.

[58] Rispo A, Williams ID, Shaw PJ (2015). Source Segregation and food waste prevention activities in high density households in a deprived urban area. Waste Manag.

[59] Roca-Barcelo A, Douglas P, Fechta D, Sterrantino AF, Williams B, Blangiardo M, Gullivere J, Hayes ET (2020). Hansell AL (2020) Risk of respiratory hospital admission associated with modelled concentrations of *Aspergillus fumigatus* from composting facilities in England. Environ Res.

[60] Rodić L, Wilson DC (2017). Resolving governance issues to achieve priority sustainable development goals related to solid waste management in developing countries. Sustainability.

[61] Saat NZM, Hanawi SA, Subhi N, Zulfakar SS, Wahab MIA (2018). Practice and attitude on household waste management in Tumpat and Kuala Krai, Kelantan. Res J Social Sci.

[62] Samah MAA, Manaf LA, Ahsan A, Sulaiman WNA, Agamuthu P, D'Silva JL (2013). Household Solid Waste Composition in Balakong City, Malaysia: Trend and Management. Pol J Environ Stud.

[63] Sarkodie SA, Owusu PA (2021). Impact of COVID-19 pandemic on waste management. Environ Dev Sustain.

[64] Saphores JDM, Ogunseitan OA, Shapiro AA. Willingness to engage in a proenvironmental behavior: an analysis of e-waste recycling based on a national survey of U.S. households. Resour Conserv Recycl. 2012;60:49e63.

[65] Sharma HB, Vanapalli KR, Cheela VRS, Ranjan VP, Jaglan AK, Dubey B, Goel S, Bhattacharya J (2020). Challenges, opportunities, and innovations for effective solid waste management during and post COVID-19 pandemic. Resour Conserv Recycl.

[66] Shigeru M (2011). Waste separation at home: Are Japanese municipal curbside recycling policies efficient?. Resour Conserv Recycl.

[67] Sujauddin M, Huda SMS, Hoque AR (2008). Household solid waste characteristics and management in Chittagong. Bangladesh Waste management.

[68] Suleman Y, Darko ET, Agyemang-Duah W. Solid Waste Disposal and Community Health Implications in Ghana: Evidence from Sawaba, Asokore Mampong Municipal Assembly. J Civil Environ Eng. 2015;202. 10.4172/2165-784X.1000202.

[69] Tekler ZD, Low R, Chung SY, Low JSC, Blessing L (2019). A Waste Management Behavioural Framework of Singapore’s Food Manufacturing Industry using Factor Analysis. Procedia CIRP.

[70] Tot B, Srđević B, Vujić B, Russo MAT, Vujić G (2016). Evaluation of key driver categories influencing sustainable waste management development with the analytic hierarchy process (AHP): Serbia example. Waste Manag Res.

[71] Tripathi A, Tyagi VK, Vivekanand V, Bose P, Suthar S (2020). Challenges, opportunities and progress in solid waste management during COVID-19 pandemic. Case Stud Chem Environ Eng.

[72] Van Fan Y, Jiang P, Hemzal M, Klemeš JJ (2021). An update of COVID-19 influence on waste management. Sci Total Environ.

[73] White KM, Hyde MK. The role of self-perceptions in the prediction of household recycling behavior in Australia. Environ Behav. 2012;44:785e99.

[74] Yang H, Ma M, Thompson JR, Flower RJ (2018). Waste management, informal recycling, environmental pollution and public health. J Epidemiol Community Health.

[75] Yatim SRM, Arshad MA (2010). Household Solid Waste Characteristics and Management in Low Cost Apartment in Petaling Jaya. Selangor Health Environ J.

[76] Zhou X, Yang J, Xu S, Wang J, Zhou Q, Li Y, Tong X (2020). Rapid in-situ composting of household food waste. Process Saf Environ Prot.

[77] Ziraba AK, Haregu TN, Mberu B (2016). A review and framework for understanding the potential impact of poor solid waste management on health in developing countries. Arch Public Heal.

