# Supplementary material for: Household solid waste management practices and perceptions among residents in the East Coast of Malaysia
Source: BMC Public Health. 2022 Jan 5;22:1. doi: 10.1186/s12889-021-12274-7 (PMC8727079; doi:10.1186/s12889-021-12274-7)
Supplement: Supplementary file 1 — Additional file 1. [file 12889_2021_12274_MOESM1_ESM.pdf]

Questionnaire (English Version)

QUESTIONNAIRE

Community Based Assessment on Household Management of Solid Waste among Residents in Kota Bharu district, Kelantan.

Instructions:

1. This questionnaire composed of 2 parts which are:

PART 1: SOCIO-DEMOGRAPHIC DATA
PART 2: PRACTICES OF SOLID WASTE MANAGEMENT AT HOUSEHOLD LEVEL

2. Participant must answer all the questions provided.

3. Please fill in the blanks or circle the desired answers based on the type of question given.

NOTES

All information provided is confidential. Data obtained from this study is used solely for the purpose of this research only.

Part 1: SOCIO-DEMOGRAPHIC DATA

Please fill (×) in the blanks with the appropriate answer.

1.1 Gender

Male ☐

Female ☐

1.2 Age

18 – 24 ☐

25 – 29 ☐

30 – 34 ☐

35 - 49 ☐

1.3 Educational Level

Primary School Education ☐

Secondary School Education ☐

Certificate/Diploma ☐

Undergraduate Education ☐

Postgraduate Degree ☐

Other: ___________________________

1.4 Marital Status

Single ☐

Married ☐

Divorced ☐

1.5 Religion

Muslim ☐

Buddha ☐

Christian ☐

Other: ________

1.6 Average monthly household income

<RM1K ☐

RM1-2K ☐

RM2-3K ☐

RM4-5K ☐

RM5-10K ☐

>RM10K ☐

1.7 Occupation

- Self-employed ☐
- Private-sector employed ☐
- Housewife ☐
- Civil servant ☐
- Retiree ☐
- Other: ________________

1.8 Type of housing unit

- Bungalow ☐
- Detached house ☐
- Semi-detached house ☐
- Other (specify):_______________

1.9 Number of people living in the house

- 1 – 3 ☐
- 4 – 6 ☐
- >7 ☐

1.10 Cook at home

- No ☐
- Yes ☐

1.11 Cooking frequency

- Daily ☐
- Two times a week ☐
- Three times a week ☐
- Once a week ☐
- Not cooking ☐

Part 2: HOUSEHOLD SOLID WASTE MANAGEMENT PRACTICES

METHODS OF WASTE DISPOSAL

2.1 What are the types of waste generated by household?

Food debris ☐ Bottles and cans ☐

Plastics ☐ Others (Specify):___________

2.2 Separation of household waste

Yes ☐

No ☐

2.3 Where do you dispose your household waste?

Appropriate site ☐

Inappropriate site ☐

2.4 Who dispose the waste to the nearest bin provided by the local authority?

Own self ☐ Paid collector ☐

Children ☐ Others ☐

PERCEPTION OF HOUSEHOLD TOWARD WASTE MANAGEMENT

2.5 Do you think waste management is important?

It is important ☐

It is not important ☐

2.6 Who is responsible to clean waste in the residential area?

Residence ☐ District council ☐

Community ☐ Private waste operators ☐

2.7 Can poor waste management contribute to disease occurrence?

Yes ☐

No ☐

Not sure ☐

2.8 What are the kinds of disease it can cause?

Malaria ☐ Diarrhea ☐

Typhoid ☐ Others ☐

2.9 Do you educate your household on proper waste disposal?

Yes ☐

No ☐

2.10 How do you motivate your household to dispose their waste?

Cleanliness ☐

Fear of illness ☐

Odour ☐
