# Supplementary material for: Household solid waste management practices and perceptions among residents in the East Coast of Malaysia
Source: BMC Public Health. 2022 Jan 5;22:1. doi: 10.1186/s12889-021-12274-7 (PMC8727079; doi:10.1186/s12889-021-12274-7)
Supplement: Supplementary file 2 — Additional file 2. [file 12889_2021_12274_MOESM2_ESM.pdf]

Supplementary material

Household Solid Waste Management Practices and Perceptions Among Residents in The East Coast of Malaysia

Widad Fadhullah; Nor Iffah Najwa Imran; Sharifah Norkhadijah Syed Ismail; Mohd Hafiidz Jaafar; Hasmah Abdullah

BMC Public Health

SOCIO-DEMOGRAPHIC CHARACTERISTICS OF STUDY PARTICIPANTS

ID	**Gender**	Age	Education	Marital Status	Religion	Monthly earnings	Occupation	Type of housing unit	Number of people living in the house	Cooking at home	Cooking frequency
1	male	35-49	Diploma/Degree	married	muslim	rm2-3k	self-employed	village	4-6 people	yes	daily
2	male	35-49	Diploma/Degree	married	muslim	rm4-5k	civil servant	bungalow	4-6 people	yes	daily
3	female	25-29	Diploma/Degree	single	muslim	rm1-2k	private-sector employed	bungalow	4-6 people	yes	daily
4	female	35-49	Secondary/Certificate	divorced	muslim	<rm1k	private-sector employed	village	1-3 people	yes	three times a week
5	male	30-34	Diploma/Degree	married	muslim	rm4-5k	civil servant	detached house	4-6 people	no	not cooking
6	female	35-49	Secondary/Certificate	divorced	muslim	<rm1k	self-employed	village	4-6 people	yes	daily
7	female	50-65	Secondary/Certificate	married	muslim	<rm1k	housewife	village	4-6 people	yes	daily
8	male	30-34	Secondary/Certificate	married	muslim	<rm1k	self-employed	village	1-3 people	yes	daily
9	male	50-65	Secondary/Certificate	married	muslim	rm2-3k	civil servant	village	4-6 people	yes	three times a week

10	male	35-49	Diploma/Degree	married	muslim	rm4-5k	civil servant	village	4-6 people	yes	daily
11	male	50-65	Secondary/Certificate	married	muslim	rm1-2k	civil servant	village	4-6 people	yes	daily
12	female	50-65	Secondary/Certificate	married	muslim	rm1-2k	private-sector employed	village	4-6 people	yes	2 times a week
13	female	30-34	Secondary/Certificate	married	muslim	<rm1k	others	village	4-6 people	yes	daily
14	female	35-49	Secondary/Certificate	married	muslim	<rm1k	self-employed	village	4-6 people	yes	daily
15	female	50-65	Secondary/Certificate	married	muslim	rm2-3k	retiree	village	4-6 people	yes	daily
16	male	50-65	Secondary/Certificate	married	muslim	<rm1k	others	village	1-3 people	yes	daily
17	female	50-65	Secondary/Certificate	married	muslim	<rm1k	self-employed	village	4-6 people	yes	daily
18	female	50-65	primary	married	muslim	rm1-2k	housewife	village	1-3 people	yes	three times a week
19	male	>65	Secondary/Certificate	married	muslim	<rm1k	self-employed	bungalow	1-3 people	yes	three times a week
20	female	50-65	Secondary/Certificate	married	muslim	rm5-10k	housewife	bungalow	1-3 people	yes	daily
21	female	35-49	Secondary/Certificate	married	muslim	rm1-2k	self-employed	village	>6	yes	daily
22	female	50-65	Secondary/Certificate	married	muslim	<rm1k	self-employed	village	>6	yes	daily
23	male	50-65	Diploma/Degree	married	muslim	<rm1k	self-employed	village	>6	yes	daily
24	female	35-49	Secondary/Certificate	married	muslim	<rm1k	housewife	village	4-6 people	yes	daily

25	male	18-24	Secondary/Certificate	single	muslim	rm1-2k	self-employed	village	>6	yes	three times a week
26	female	50-65	Secondary/Certificate	married	muslim	<rm1k	housewife	village	>6	yes	daily
27	male	50-65	Diploma/Degree	married	muslim	rm1-2k	private-sector employed	bungalow	4-6 people	yes	three times a week
28	female	>68	primary	married	muslim	<rm1k	others	village	1-3 people	yes	three times a week
29	male	30-34	Secondary/Certificate	single	muslim	<rm1k	self-employed	village	1-3 people	no	not cooking
30	female	18-24	Diploma/Degree	single	muslim	rm2-3k	others	village	4-6 people	yes	daily
31	male	18-24	Diploma/Degree	single	muslim	rm2-3k	private-sector employed	Others	>6	Yes	daily
32	female	50-65	Secondary/Certificate	married	muslim	rm1-2k	housewife	Others	1-3 people	Yes	three times a week
33	male	30-34	Diploma/Degree	married	muslim	rm2-3k	retiree	bungalow	1-3 people	No	not cooking
34	male	50-65	Secondary/Certificate	married	buddha	rm2-3k	self-employed	Others	4-6 people	No	not cooking
35	female	35-49	Secondary/Certificate	married	muslim	<rm1k	housewife	Others	4-6 people	Yes	daily
36	female	35-49	Secondary/Certificate	married	buddha	rm4-5k	housewife	detached house	4-6 people	Yes	daily
37	male	50-65	Secondary/Certificate	married	muslim	rm1-2k	retiree	Others	4-6 people	Yes	daily
38	male	30-34	Diploma/Degree	married	muslim	rm2-3k	private-sector employed	Others	4-6 people	Yes	daily

39	male	50-65	Secondary/Certificate	married	muslim	rm1-2k	self-employed	Others	4-6 people	Yes	three times a week
40	female	35-49	Diploma/Degree	married	muslim	rm5-10k	civil servant	bungalow	4-6 people	No	not cooking
41	male	35-49	Diploma/Degree	single	muslim	rm1-2k	self-employed	semi-detached house	1-3 people	No	not cooking
42	male	50-65	Secondary/Certificate	married	buddha	rm4-5k	retiree	bungalow	4-6 people	No	not cooking
43	male	35-49	Secondary/Certificate	single	muslim	<rm1k	self-employed	Others	1-3 people	Yes	daily
44	male	18-24	Diploma/Degree	single	muslim	<rm1k	private-sector employed	Others	1-3 people	Yes	daily
45	female	30-34	Diploma/Degree	married	muslim	rm5-10k	civil servant	Others	4-6 people	Yes	three times a week
46	male	30-34	Diploma/Degree	married	muslim	rm2-3k	civil servant	Others	4-6 people	No	not cooking
47	female	50-65	Secondary/Certificate	married	muslim	rm1-2k	housewife	Others	1-3 people	Yes	three times a week
48	female	50-65	Secondary/Certificate	married	muslim	<rm1k	self-employed	detached house	>6	No	not cooking
49	male	50-65	Secondary/Certificate	married	muslim	rm2-3k	retiree	Others	1-3 people	Yes	once a week
50	female	35-49	Diploma/Degree	married	muslim	rm4-5k	civil servant	Others	4-6 people	Yes	daily
51	female	50-65	Secondary/Certificate	married	muslim	rm4-5k	housewife	Others	4-6 people	Yes	daily
52	female	50-65	Primary	married	buddha	rm1-2k	housewife	Others	4-6 people	Yes	daily

53	female	18-24	Secondary/Certificate	single	buddha	rm2-3k	self-employed	Others	>6	Yes	three times a week
54	male	50-65	Diploma/Degree	married	muslim	rm5-10k	retiree	bungalow	1-3 people	No	not cooking
55	female	50-65	Postgraduate	married	muslim	rm5-10k	housewife	bungalow	>6	Yes	daily
56	female	25-29	Secondary/Certificate	married	muslim	rm1-2k	housewife	bungalow	4-6 people	Yes	three times a week
57	male	30-34	Secondary/Certificate	married	buddha	rm5-10k	self-employed	bungalow	4-6 people	Yes	daily
58	female	18-24	Secondary/Certificate	single	muslim	<rm1k	student	Others	4-6 people	Yes	three times a week
59	female	50-65	Secondary/Certificate	married	muslim	rm2-3k	housewife	Others	4-6 people	Yes	daily
60	male	35-49	Secondary/Certificate	married	muslim	rm4-5k	civil servant	Others	4-6 people	Yes	three times a week
61	female	25-29	Diploma/Degree	married	muslim	rm2-3k	civil servant	detached house	4-6 people	Yes	three times a week
62	male	50-65	Secondary/Certificate	married	muslim	rm1-2k	self-employed	others	4-6 people	Yes	daily
63	male	35-49	Diploma/Degree	married	muslim	rm2-3k	private-sector employed	bungalow	4-6 people	Yes	daily
64	female	30-34	Secondary/Certificate	single	muslim	rm1-2k	housewife	others	4-6 people	No	not cooking
65	male	30-34	Postgraduate	single	muslim	rm5-10k	civil servant	bungalow	1-3 people	Yes	daily
66	male	50-65	Secondary/Certificate	married	muslim	rm1-2k	self-employed	detached house	4-6 people	Yes	every other day

67	female	18-24	Primary	married	muslim	rm2-3k	housewife	detached house	4-6 people	Yes	daily
68	male	25-29	Diploma/Degree	married	muslim	rm1-2k	civil servant	detached house	1-3 people	Yes	three times a week
69	female	35-49	Diploma/Degree	married	muslim	rm1-2k	civil servant	others	4-6 people	Yes	daily
70	male	25-29	Diploma/Degree	married	muslim	rm4-5k	private-sector employed	others	4-6 people	Yes	three times a week
71	male	35-49	Diploma/Degree	married	muslim	rm5-10k	civil servant	others	>6	Yes	daily
72	male	50-65	Secondary/Certificate	married	muslim	rm2-3k	retiree	others	4-6 people	Yes	three times a week
73	male	>65	Primary	married	muslim	rm1-2k	retiree	others	4-6 people	Yes	daily
74	female	35-49	Diploma/Degree	married	muslim	rm2-3k	self-employed	bungalow	4-6 people	Yes	once a week
75	female	35-49	Secondary/Certificate	married	muslim	rm2-3k	civil servant	others	4-6 people	No	not cooking
76	male	35-49	Diploma/Degree	married	muslim	rm5-10k	civil servant	bungalow	>6	Yes	once a week
77	male	>65	Secondary/Certificate	married	muslim	rm2-3k	retiree	others	4-6 people	Yes	daily
78	female	25-29	Diploma/Degree	married	muslim	rm2-3k	private-sector employed	semi-detached house	1-3 people	No	not cooking
79	female	50-65	Secondary/Certificate	married	muslim	rm2-3k	self-employed	others	4-6 people	Yes	daily
80	female	30-34	Secondary/Certificate	married	muslim	rm1-2k	self-employed	others	4-6 people	Yes	three times a week
81	male	50-65	Secondary/Certificate	married	buddha	rm4-5k	self-employed	bungalow	>6	Yes	daily

82	male	35-49	Secondary/Certificate	married	buddha	rm5-10k	self-employed	semi-detached house	4-6 people	No	not cooking
83	male	50-65	Secondary/Certificate	married	muslim	rm1-2k	self-employed	others	4-6 people	No	not cooking
84	female	25-29	Diploma/Degree	married	muslim	rm1-2k	private-sector employed	others	4-6 people	Yes	daily
85	female	50-65	Primary	married	muslim	<rm1k	housewife	others	4-6 people	Yes	three times a week
86	female	25-29	Diploma/Degree	married	muslim	<rm1k	housewife	detached house	4-6 people	Yes	daily
87	male	25-29	Diploma/Degree	single	muslim	rm2-3k	self-employed	others	>6	Yes	daily
88	female	18-24	Diploma/Degree	single	muslim	rm1-2k	student	others	4-6 people	Yes	daily
89	male	35-49	Secondary/Certificate	married	muslim	rm2-3k	self-employed	others	4-6 people	Yes	daily
90	female	35-49	Diploma/Degree	married	muslim	rm2-3k	civil servant	others	4-6 people	Yes	daily
91	female	18-24	Secondary/Certificate	married	muslim	<rm1k	housewife	terrace	4-6 people	yes	daily
92	male	50-65	Primary	married	muslim	rm2-3k	self-employed	terrace	1-3 people	yes	daily
93	male	50-65	Secondary/Certificate	married	muslim	<rm1k	retiree	village	4-6 people	yes	daily
94	male	35-49	Secondary/Certificate	married	muslim	rm1-2k	self-employed	village	4-6 people	yes	daily
95	female	50-65	Secondary/Certificate	married	muslim	<rm1k	housewife	semi-detached house	1-3 people	yes	daily
96	female	50-58	Secondary/Certificate	married	muslim	rm1-2k	housewife	village	4-6 people	yes	daily

97	male	18-24	Diploma/Degree	single	muslim	rm2-3k	self-employed	village	4-6 people	yes	daily
98	female	25-29	Secondary/Certificate	married	muslim	rm1-2k	self-employed	village	4-6 people	yes	2 times a week
99	female	30-34	Diploma/Degree	married	muslim	rm2-3k	private-sector employed	semi-detached house	1-3 people	yes	daily
100	female	25-29	Secondary/Certificate	married	muslim	<rm1k	housewife	terrace	4-6 people	yes	daily
101	female	25-29	Secondary/Certificate	married	muslim	rm2-3k	self-employed	village	4-6 people	yes	daily
102	female	35-49	Secondary/Certificate	married	muslim	rm2-3k	housewife	terrace	4-6 people	yes	daily
103	female	30-34	Secondary/Certificate	married	muslim	<rm1k	others	village	4-6 people	yes	daily
104	female	30-34	Diploma/Degree	married	muslim	rm4-5k	civil servant	terrace	4-6 people	yes	daily
105	male	18-24	Diploma/Degree	single	muslim	rm1-2k	student	village	4-6 people	yes	daily
106	male	50-65	Secondary/Certificate	married	muslim	<rm1k	others	village	1-3 people	yes	daily
107	male	25-29	Secondary/Certificate	single	muslim	rm1-2k	self-employed	village	>6	yes	daily
108	female	50-65	primary	married	muslim	rm1-2k	housewife	village	1-3 people	yes	three times a week
109	female	18-24	Secondary/Certificate	single	muslim	<rm1k	self-employed	village	1-3 people	yes	three times a week
110	female	18-24	Secondary/Certificate	married	muslim	<rm1k	housewife	detached house	4-6 people	yes	daily
111	female	18-24	Diploma/Degree	single	muslim	<rm1k	others	village	1-3 people	yes	daily

112	male	35-49	Diploma/Degree	single	muslim	rm5-10k	self-employed	bungalow	4-6 people	yes	three times a week
113	male	50-65	Diploma/Degree	married	muslim	<rm1k	self-employed	village	>6	yes	daily
114	female	>65	primary	married	muslim	<rm1k	housewife	village	1-3 people	yes	daily
115	male	18-24	Secondary/Certificate	single	muslim	rm1-2k	self-employed	village	>6	yes	three times a week
116	male	35-49	postgraduate	married	muslim	rm5-10k	private-sector employed	bungalow	4-6 people	yes	daily
117	male	50-65	Diploma/Degree	married	muslim	rm1-2k	private-sector employed	bungalow	4-6 people	yes	three times a week
118	male	18-24	Secondary/Certificate	single	muslim	rm1-2k	student	bungalow	>6	yes	daily
119	male	30-34	Secondary/Certificate	single	muslim	<rm1k	self-employed	village	1-3 people	no	not cooking
120	male	35-49	Diploma/Degree	married	muslim	rm4-5k	self-employed	bungalow	4-6 people	yes	daily
121	male	18-24	Secondary/Certificate	single	muslim	<rm1k	student	bungalow	4-6 people	no	not cooking
122	male	25-29	Secondary/Certificate	single	muslim	<rm1k	private-sector employed	bungalow	4-6 people	yes	daily
123	female	35-49	Secondary/Certificate	married	muslim	rm1-2k	self-employed	bungalow	4-6 people	yes	daily
124	female	50-65	Secondary/Certificate	single	muslim	rm1-2k	housewife	bungalow	1-3 people	yes	three times a week
125	female	18-24	Diploma/Degree	married	muslim	<rm1k	housewife	terrace	1-3 people	yes	daily

126	female	50-65	Secondary/Certificate	married	muslim	rm4-5k	private-sector employed	terrace	4-6 people	yes	2 times a week
127	female	25-29	Diploma/ Degree	married	muslim	rm5-10k	civil servant	semi-detached house	1-3 people	yes	daily
128	male	35-49	Secondary/Certificate	married	muslim	rm5-10k	civil servant	bungalow	>6	yes	once a week
129	female	35-49	Secondary/Certificate	married	muslim	rm1-2k	housewife	village	1-3 people	yes	daily
130	female	35-49	Secondary/Certiifcate	married	muslim	<rm1k	housewife	detached house	4-6 people	yes	daily
131	female	30-34	Secondary/Certificate	married	muslim	rm1-2k	self-employed	terrace	4-6 people	yes	daily
132	female	30-34	Diploma/Degree	married	muslim	<rm1k	housewife	detached house	4-6 people	yes	daily
133	female	30-34	Secondary/Certiifcate	married	muslim	rm1-2k	self-employed	terrace	4-6 people	yes	daily
134	male	>65	primary	married	muslim	rm1-2k	retiree	terrace	1-3 people	yes	daily
135	male	30-34	Diploma/Degree	married	muslim	rm2-3k	private-sector employed	village	4-6 people	yes	daily
136	male	25-29	Diploma/Degree	single	muslim	rm1-2k	self-employed	village	4-6 people	yes	daily
137	female	35-49	Secondary/Certificate	married	muslim	rm1-2k	private-sector employed	semi-detached house	>6	yes	daily
138	female	30-34	Diploma/Degree	married	muslim	rm2-3k	private-sector employed	terrace	4-6 people	yes	2 times a week
139	female	35-49	primary	married	muslim	<rm1k	housewife	terrace	4-6 people	yes	daily
140	female	50-65	Secondary/Certificate	married	muslim	rm2-3k	housewife	terrace	1-3 people	yes	daily

141	female	35-49	Secondary/Certificate	married	muslim	rm2-3k	housewife	terrace	4-6 people	yes	daily
142	female	30-34	Secondary/Certificate	married	muslim	rm1-2k	housewife	terrace	4-6 people	yes	daily
143	female	35-49	Secondary/Certificate	married	muslim	rm5-10k	civil servant	terrace	4-6 people	yes	daily
144	female	30-34	Secondary/Certificate	married	muslim	<rm1k	housewife	terrace	4-6 people	yes	daily
145	male	35-49	primary	married	muslim	rm2-3k	self-employed	terrace	4-6 people	yes	daily
146	female	25-29	Diploma/Degree	married	muslim	rm5-10k	private-sector employed	terrace	4-6 people	yes	three times a week
147	female	25-29	Diploma/Degree	single	muslim	rm2-3k	private-sector employed	terrace	4-6 people	yes	three times a week
148	male	25-29	Secondary/Certificate	single	muslim	<rm1k	self-employed	terrace	1-3 people	no	not cooking
149	male	25-29	Secondary/Certificate	married	muslim	rm1-2k	self-employed	terrace	1-3 people	yes	daily
150	male	30-34	Secondary/Certificate	married	muslim	rm2-3k	self-employed	terrace	4-6 people	yes	daily
151	female	35-49	Secondary/Certificate	married	muslim	rm2-3k	housewife	terrace	4-6 people	yes	daily
152	female	30-34	Secondary/Certificate	married	muslim	rm1-2k	housewife	terrace	>6	yes	daily
153	male	18-24	Secondary/Certificate	single	muslim	rm2-3k	self-employed	village	4-6 people	yes	daily
154	male	35-49	Secondary/Certificate	married	muslim	rm4-5k	self-employed	terrace	4-6 people	yes	daily
155	female	30-34	Secondary/Certificate	married	muslim	rm1-2k	self-employed	terrace	4-6 people	yes	daily
156	male	35-49	Secondary/Certificate	married	muslim	rm4-5k	self-employed	bungalow	1-3 people	yes	daily

157	male	18-24	Diploma/Degree	married	muslim	rm2-3k	self-employed	semi-detached house	4-6 people	no	not cooking
158	female	35-49	Secondary/Certificate	married	muslim	<rm1k	housewife	terrace	>6	yes	daily
159	male	>56	Secondary/Certificate	married	muslim	rm2-3k	civil servant	terrace	1-3 people	yes	three times a week
160	female	25-29	Secondary/Certificate	married	muslim	rm1-2k	housewife	terrace	4-6 people	yes	daily
161	male	30-34	Secondary/Certificate	married	muslim	rm2-3k	civil servant	terrace	4-6 people	yes	daily
162	male	>60	Secondary/Certificate	married	muslim	rm4-5k	retiree	terrace	1-3 people	yes	2 times a week
163	male	39-49	Secondary/Certificate	married	muslim	rm2-3k	private-sector employed	terrace	4-6 people	yes	three times a week
164	female	>56	Secondary/Certificate	married	muslim	rm2-3k	housewife	terrace	>6	yes	daily
165	female	25-29	Secondary/Certiifcate	married	muslim	rm2-3k	housewife	semi-detached house	1-3 people	yes	daily
166	female	25-29	Secondary/Certificate	married	muslim	rm1-2k	housewife	detached house	1-3 people	yes	daily
167	female	18-24	Secondary/Certificate	married	muslim	>rm1k	housewife	semi-detached house	4-6 people	yes	three times a week
168	female	25-29	Secondary/Certificate	married	muslim	rm1-2k	private-sector employed	terrace	4-6 people	yes	daily
169	male	35-49	Secondary/Certificate	single	muslim	rm2-3k	private-sector employed	detached house	4-6 people	yes	three times a week
170	female	>59	Secondary/Certificate	married	muslim	>rm1k	housewife	detached house	4-6 people	yes	daily

171	female	25-29	Secondary/Certificate	married	muslim	>rm1k	housewife	terrace	1-3 people	yes	daily
172	male	25-29	Secondary/Certificate	married	muslim	rm1-2k	civil servant	semi-detached house	4-6 people	yes	daily
173	male	35-49	Secondary/Certificate	married	muslim	rm2-3k	civil servant	terrace	4-6 people	yes	daily
174	male	35-49	Diploma/Degree	married	muslim	rm2-3k	civil servant	semi-detached house	4-6 people	yes	2 times a week
175	female	18-24	Diploma/Degree	married	muslim	rm2-3k	private-sector employed	detached house	4-6 people	yes	daily
176	female	35-49	Secondary/Certificate	single	muslim	>rm1k	self-employed	terrace	4-6 people	yes	daily
177	female	>57	Secondary/Certificate	married	muslim	>rm1k	retiree	terrace	1-3 people	yes	daily
178	female	18-24	Diploma/Degree	single	muslim	>rm1k	self-employed	semi-detached house	4-6 people	yes	daily
179	male	35-49	Secondary/Certificate	married	muslim	rm2-3k	self-employed	terrace	4-6 people	yes	2 times a week
180	male	35-49	Secondary/Certificate	married	muslim	rm4-5k	civil servant	terrace	>6	yes	daily
181	male	35-49	Secondary/Certificate	married	muslim	rm1-2k	private-sector employed	bungalow	4-6 people	yes	three times a week
182	female	>61	Secondary/Certificate	married	muslim	>rm1k	housewife	bungalow	4-6 people	yes	daily
183	male	30-34	Diploma/Degree	married	muslim	rm2-3k	civil servant	detached house	1-3 people	yes	daily
184	male	35-49	Diploma/Degree	married	muslim	rm4-5k	civil servant	semi-detached house	4-6 people	yes	2 times a week
185	female	18-24	Diploma/Degree	single	muslim	rm5-10k	self-employed	terrace	4-6 people	yes	2 times a week

186	female	30-34	Secondary/Certificate	married	muslim	rm2-3k	self-employed	detached house	>6	yes	daily
187	male	25-29	Diploma/Degree	single	muslim	rm1-2k	self-employed	semi-detached house	1-3 people	no	not cooking
188	male	25-29	Secondary/Certificate	single	muslim	>rm1k	self-employed	semi-detached house	1-3 people	yes	2 times a week
189	female	>52	Secondary/Certificate	married	muslim	rm1-2k	housewife	semi-detached house	1-3 people	no	not cooking
190	male	30-34	Secondary/Certificate	single	muslim	rm1-2k	private-sector employed	village	>6	yes	daily
191	male	>62	Secondary/Certificate	married	muslim	>rm1k	housewife	terrace	4-6 people	yes	daily
192	female	>54	Secondary/Certificate	married	muslim	rm2-3k	housewife	terrace	4-6 people	yes	daily
193	male	30-34	Secondary/Certificate	married	muslim	rm1-2k	self-employed	terrace	4-6 people	no	not cooking
194	female	30-34	Secondary/Certificate	married	muslim	rm2-3k	self-employed	village	4-6 people	yes	2 times a week
195	female	35-49	Secondary/Certiifcate	married	muslim	>rm1k	self-employed	village	4-6 people	yes	2 times a week
196	male	35-49	Secondary/Certificate	married	muslim	rm1-2k	private-sector employed	detached house	1-3 people	yes	2 times a week
197	female	25-29	Secondary/Certificate	married	muslim	>rm1k	private-sector employed	Detached house	>6	yes	daily
198	female	>62	Diploma/Degree	married	muslim	rm2-3k	retiree	bungalow	>6	yes	daily
199	female	>58	primary school	married	muslim	rm1-2k	civil servant	village	1-3 people	yes	daily
200	male	25-29	Secondary/Certificate	married	muslim	rm1-2k	self-employed	detached house	>6	yes	2 times a week
201	female	35-49	Secondary/Certificate	married	muslim	>rm1k	self-employed	detached house	4-6 people	yes	2 times a week

202	female	35-49	Secondary/Certificate	married	muslim	rm2-3k	private-sector employed	terrace	4-6 people	yes	2 times a week
203	female	35-49	Secondary/Certificate	married	muslim	rm2-3k	private-sector employed	bungalow	4-6 people	yes	daily
204	female	>62	Secondary/Certificate	married	muslim	>rm1k	retiree	village	1-3 people	yes	2 times a week
205	female	35-49	Diploma/Degree	married	muslim	rm2-3k	housewife	detached house	>6	yes	daily
206	female	>51	primary	married	muslim	>rm1k	private-sector employed	village	1-3 people	yes	daily
207	male	25-29	Secondary/Certificate	married	muslim	rm1-2k	self-employed	semi-detached house	1-3 people	yes	daily
208	female	35-49	Secondary/Certificate	married	muslim	>rm1k	self-employed	detached house	4-6 people	yes	2 times a week
209	female	18-24	Diploma/Degree	single	muslim	rm2-3k	self-employed	terrace	4-6 people	yes	2 times a week
210	female	30-34	Secondary/Certificate	married	muslim	rm2-3k	self-employed	detached house	>6	yes	daily
211	female	30-34	Secondary/Certificate	married	muslim	rm2-3k	self-employed	village	4-6 people	yes	2 times a week
212	male	35-49	Secondary/Certificate	married	muslim	rm2-3k	civil servant	terrace	4-6 people	yes	2 times a week
213	male	35-49	Secondary/Certificate	married	muslim	rm4-5k	self-employed	terrace	>6	yes	daily
214	female	20-29	Diploma/Degree	married	muslim	rm4-5k	housewife	bungalow	4-6 people	yes	daily
215	female	35-49	Secondary/Certificate	married	muslim	rm2-3k	housewife	terrace	4-6 people	yes	daily
216	male	50-65	Secondary/Certificate	divorced	muslim	rm2-3k	Private-sector employed	Bungalow	1-3 people	No	not cooking

217	Female	30-34	Diploma/Degree	Married	muslim	rm1-2k	private-sector employed	Other	1-3 people	Yes	Daily
218	male	30-34	Diploma/Degree	Married	muslim	rm2-3k	Private-sector employed	Other	4-6 people	Yes	Daily
219	male	30-34	Diploma/Degree	Married	muslim	rm2-3k	Private-sector employed	Other	4-6 people	Yes	Daily
220	male	50-65	Secondary/Certificate	Married	muslim	rm2-3k	retiree	Other	1-3 people	Yes	Three times a week
221	Female	50-65	Secondary/Certificate	Married	muslim	rm2-3k	retiree	Other	1-3 people	Yes	Three times a week
222	Female	35-49	Secondary/Certificate	Married	muslim	<rm1k	Self-employed	Other	4-6 people	Yes	Daily
223	Female	50-65	Primary	Single	muslim	<rm1k	Housewife	Other	4-6 people	Yes	Daily
224	Female	35-49	Diploma/Degree	Married	muslim	rm4-5k	Civil servant	Bungalow	4-6 people	Yes	Daily
225	Female	50-65	Secondary/Certificate	Married	muslim	<rm1k	Self-employed	Bungalow	1-3 people	Yes	Daily
226	male	50-65	Secondary/Certificate	Married	muslim	<rm1k	Self-employed	Bungalow	4-6 people	Yes	Daily
227	Female	30-34	Diploma/Degree	Married	muslim	rm2-3k	Housewife	Bungalow	1-3 people	Yes	Daily
228	Female	35-49	Secondary/Certificate	Married	muslim	rm1-2k	Self-employed	Bungalow	4-6 people	Yes	Daily
229	Female	>65	other	Single	muslim	<rm1k	Self-employed	Bungalow	1-3 people	Yes	Daily
230	male	50-65	Diploma/Degree	Married	muslim	rm4-5k	retiree	Bungalow	4-6 people	Yes	Daily

231	Female	30-34	Diploma/Degree	Married	muslim	rm2-3k	Private-sector employed	Semi-detached house	4-6 people	Yes	Daily
232	male	35-49	Secondary/Certificate	Married	muslim	rm2-3k	Self-employed	Bungalow	4-6 people	Yes	Daily
233	male	25-29	Diploma/Degree	Married	muslim	rm2-3k	Civil servant	Semi-detached house	1-3 people	Yes	Three times a week
234	Female	35-49	master	Married	muslim	rm4-5k	Civil servant	Bungalow	4-6 people	Yes	2 times a week
235	male	18-24	Secondary/Certificate	Single	muslim	<rm1k	Civil servant	Other	1-3 people	Yes	Daily
236	male	18-24	master	Single	muslim	<rm1k	Private-sector employed	Other	4-6 people	Yes	Daily
237	male	50-65	Diploma/Degree	Married	muslim	rm4-5k	Civil servant	Bungalow	4-6 people	Yes	Daily
238	Female	50-65	Secondary/Certificate	Married	muslim	<rm1k	Housewife	Other	1-3 people	Yes	Daily
239	Female	30-34	Secondary/Certificate	Married	muslim	rm1-2k	Private-sector employed	Other	4-6 people	Yes	Daily
240	male	35-49	Diploma/Degree	Married	muslim	rm2-3k	Self-employed	Bungalow	>6	Yes	Daily
241	Female	35-49	Secondary/Certificate	divorced	muslim	<rm1k	Private-sector employed	Other	>6	Yes	Daily
242	Female	35-49	Secondary/Certificate	Married	muslim	<rm1k	Housewife	Other	4-6 people	Yes	Daily
243	Female	30-34	Secondary/Certificate	Married	muslim	rm2-3k	Housewife	Other	4-6 people	Yes	Daily
244	Female	30-34	Secondary/Certificate	Single	muslim	rm1-2k	Housewife	Other	4-6 people	Yes	Daily
245	Female	30-34	Primary	Married	muslim	rm2-3k	Housewife	Other	4-6 people	Yes	Daily

246	Female	50-65	Secondary/Certificate	Married	muslim	rm2-3k	Housewife	Other	4-6 people	Yes	Daily
247	male	50-65	Secondary/Certificate	Married	muslim	rm2-3k	Self-employed	Other	4-6 people	Yes	Daily
248	male	50-65	Secondary/Certificate	Married	muslim	rm4-5k	retiree	Other	1-3 people	Yes	Daily
249	Female	25-29	Diploma/Degree	Married	muslim	rm4-5k	Housewife	Bungalow	>6	Yes	Daily
250	Female	35-49	Diploma/Degree	Single	Buddha	rm4-5k	Private-sector employed	Other	1-3 people	Yes	Daily
251	Female	35-49	Secondary/Certificate	Married	muslim	rm4-5k	Civil servant	Other	4-6 people	Yes	Daily
252	male	35-49	other	Married	muslim	>rm10k	Civil servant	Semi-detached house	4-6 people	Yes	Daily
253	Female	25-29	Secondary/Certificate	Married	muslim	rm1-2k	Self-employed	Bungalow	>6	Yes	Daily
254	male	18-24	Diploma/Degree	Single	muslim	rm1-2k	Private-sector employed	Bungalow	1-3 people	Yes	Daily
255	Female	18-24	Diploma/Degree	Single	muslim	rm1-2k	Self-employed	Other	1-3 people	Yes	Daily
256	Female	30-34	master	Married	muslim	rm4-5k	Housewife	Bungalow	4-6 people	Yes	Daily
257	Female	50-65	Secondary/Certificate	Single	muslim	rm1-2k	Housewife	Bungalow	1-3 people	Yes	Daily
258	male	35-49	Primary	Single	muslim	rm1-2k	Private-sector employed	Bungalow	>6	Yes	Daily
259	Female	25-29	Diploma/Degree	Single	muslim	<rm1k	Private-sector employed	Bungalow	4-6 people	Yes	Daily

260	Female	35-49	Secondary/Certificate	Married	muslim	rm1-2k	Private-sector employed	Other	1-3 people	Yes	Daily
261	male	30-34	Secondary/Certificate	Married	muslim	rm1-2k	Private-sector employed	Other	1-3 people	Yes	Daily
262	male	35-49	Secondary/Certificate	Single	muslim	rm1-2k	Self-employed	Other	1-3 people	Yes	Daily
263	Female	35-49	Secondary/Certificate	Married	muslim	<rm1k	Self-employed	Semi-detached house	>6	Yes	Daily
264	male	30-34	master	Married	muslim	rm4-5k	Self-employed	Semi-detached house	4-6 people	Yes	Daily
265	Female	50-65	Secondary/Certificate	Single	muslim	<rm1k	Self-employed	Other	>6	Yes	Daily
266	Female	25-29	Diploma/Degree	Single	muslim	rm4-5k	civil servant	Other	1-3 people	Yes	Three times a week
267	male	35-49	Secondary/Certificate	Married	muslim	rm1-2k	Civil servant	Other	1-3 people	Yes	Daily
268	Female	25-29	Secondary/Certificate	Married	muslim	<rm1k	Housewife	Semi-detached house	1-3 people	Yes	Daily
269	Female	35-49	Secondary/Certificate	Married	muslim	rm4-5k	Civil servant	Other	4-6 people	Yes	Daily
270	male	35-49	Secondary/Certificate	Married	muslim	<rm1k	Student	Semi-detached house	>6	Yes	Daily
271	male	>65	Secondary/Certificate	Married	muslim	rm2-3k	Self-employed	Other	1-3 people	Yes	Daily
272	Female	30-34	Diploma/Degree	Married	muslim	rm2-3k	Civil servant	Semi-detached house	4-6 people	Yes	Three times a week

273	Female	35-49	Diploma/Degree	Married	muslim	rm4-5k	Civil servant	Semi-detached house	4-6 people	Yes	Daily
274	Female	18-24	Diploma/Degree	Single	muslim	rm2-3k	Self-employed	Bungalow	>6	Yes	Daily
275	Female	18-24	Diploma/Degree	Single	muslim	rm2-3k	Self-employed	Bungalow	1-3 people	Yes	Daily
276	Female	18-24	Diploma/Degree	Single	muslim	rm4-5k	Civil servant	Bungalow	4-6 people	No	not cooking
277	Female	18-24	Diploma/Degree	Single	muslim	rm1-2k	private-sector employed	Semi-detached house	1-3 people	No	not cooking
278	Female	18-24	Diploma/Degree	Single	muslim	rm4-5k	Civil servant	Bungalow	4-6 people	Yes	Daily
279	Female	25-29	Secondary/Certificate	Married	muslim	rm2-3k	Civil servant	Bungalow	4-6 people	Yes	Daily
280	male	30-34	Diploma/Degree	Married	muslim	rm4-5k	Civil servant	Bungalow	1-3 people	Yes	Daily
281	male	25-29	Diploma/Degree	Married	muslim	rm4-5k	Civil servant	Semi-detached house	1-3 people	No	not cooking
282	male	30-34	Diploma/Degree	divorced	muslim	rm2-3k	Civil servant	Semi-detached house	1-3 people	Yes	Daily
283	male	30-34	Diploma/Degree	Single	muslim	rm1-2k	Civil servant	Semi-detached house	4-6 people	Yes	Daily
284	Female	30-34	Primary	Single	muslim	<rm1k	Private-sector employed	Bungalow	>6	Yes	Daily
285	Female	25-29	Primary	Single	muslim	<rm1k	other	Bungalow	>6	Yes	Daily
286	Female	18-24	Secondary/Certificate	Single	muslim	<rm1k	Private-sector employed	Bungalow	>6	Yes	Daily

287	Female	18-24	Secondary/Certificate	Single	muslim	<rm1k	Private-sector employed	Bungalow	>6		Yes	Daily
288	male	18-24	Secondary/Certificate	Single	muslim	<rm1k	Private-sector employed	Bungalow	>6		Yes	2 times a week
289	Female	35-49	Secondary/Certificate	Married	muslim	<rm1k	Private-sector employed	Bungalow	>6		Yes	Daily
290	male	25-29	Diploma/Degree	Single	muslim	<rm1k	Private-sector employed	Bungalow	>6		Yes	Daily
291	male	18-24	Diploma/Degree	Single	muslim	<rm1k	Self-employed	Semi-detached house	>6		Yes	Daily
292	male	35-49	Secondary/Certificate	Married	muslim	rm2-3k	Civil servant	Bungalow	>6		Yes	Daily
293	male	18-24	Diploma/Degree	Single	muslim	rm1-2k	Self-employed	Semi-detached house	>6		Yes	Daily
294	male	35-49	Secondary/Certificate	Married	muslim	<rm1k	Self-employed	Semi-detached house	4-6 people		Yes	Three times a week
295	male	18-24	Diploma/Degree	Single	muslim	<rm1k	Self-employed	Bungalow	4-6 people		Yes	Daily
296	male	30-34	Diploma/Degree	Married	muslim	rm2-3k	Civil servant	Semi-detached house	1-3 people		Yes	Daily
297	male	18-24	Diploma/Degree	Single	muslim	rm1-2k	Self-employed	Bungalow	1-3 people		Yes	Daily
298	Female	18-24	Diploma/Degree	Single	christian	rm1-2k	Private-sector employed	Bungalow	1-3 people		No	not cooking

299	Female	30-34	master	Married	Muslim	rm4-5k	Private-sector employed	Bungalow	1-3 people	Yes	Daily
300	male	35-49	Secondary/Certificate	Married	Muslim	rm1-2k	retiree	Bungalow	>6	Yes	Daily
301	male	18-24	Diploma/Degree	Single	Muslim	<rm1k	Self-employed	Bungalow	1-3 people	Yes	Daily
302	Female	18-24	Diploma/Degree	Single	Muslim	<rm1k	Private-sector employed	Semi-detached house	1-3 people	Yes	Daily
303	male	25-29	Diploma/Degree	Single	Muslim	rm1-2k	Self-employed	Other	1-3 people	Yes	Daily
304	male	30-34	master	Married	Muslim	rm4-5k	Civil servant	Bungalow	1-3 people	Yes	Daily
305	male	18-24	master	Single	Muslim	rm2-3k	Self-employed	Bungalow	1-3 people	Yes	Daily
306	Female	25	Secondary/Certificate	Married	Muslim	RM2,000	Civil servant	Other	1-3 people	Yes	Three times a week
307	male	50	Primary	Married	Muslim	RM1,000	Self-employed	Other	4-6 people	Yes	Daily
308	Female	50	Primary	Married	Muslim	RM900	Housewife	Other	1-3 people	Yes	Daily
309	male	65	Primary	Married	Muslim	RM900	Retiree	Other	4-6 people	Yes	Daily
310	Female	25	Diploma/Degree	Married	Muslim	RM900	Housewife	Semi-detached house	1-3 people	Yes	Daily
311	male	30	Secondary/ Certificate	Married	Muslim	RM900	Self-employed	Other	4-6 people	Yes	Daily
312	Female	35	Secondary/Certificate	Married	Muslim	RM1,000	Civil servant	Other	>6	Yes	Daily
313	Female	30	Diploma/Degree	Married	Muslim	RM900	Civil servant	Other	4-6 people	Yes	Daily
314	male	65	Secondary/Certificate	Married	Muslim	RM2,000	Civil servant	Bungalow	1-3 people	Yes	Daily

315	male	65	Secondary/Certificate	Married	Muslim	RM4,000	Civil servant	Other	>6	No	not cooking
316	Female	25	Secondary/Certificate	Single	Muslim	RM900	Student	Other	>6	Yes	Daily
317	male	65	Primary	Married	Muslim	RM1,000	Self-employed	Other	>6	Yes	Daily
318	male	65	Diploma/Degree	Married	Muslim	RM5,000	Civil servant	Bungalow	4-6 people	Yes	Daily
319	male	35	Secondary/Certificate	Married	Muslim	RM2,000	Private-sector employed	Other	>6	Yes	Daily
320	male	35	Secondary/Certificate	Married	Muslim	RM1,000	Retiree	Other	4-6 people	Yes	Daily
321	male	25	Secondary/Certificate	Single	Muslim	RM4,000	Civil servant	Bungalow	4-6 people	Yes	Daily
322	Female	30	Diploma/Degree	Married	Muslim	RM2,000	Self-employed	Other	4-6 people	Yes	Daily
323	Female	30	Diploma/Degree	Married	Muslim	RM2,000	Civil servant	Other	4-6 people	Yes	once a week
324	Female	35	Secondary/Certificate	Married	Muslim	RM1,000	Civil servant	Other	1-3 people	Yes	Daily
325	male	35	Secondary/Certificate	Married	Muslim	RM1,000	Self-employed	Other	1-3 people	Yes	Three times a week
326	Female	65	Secondary/Certificate	Married	Muslim	RM1,000	Civil servant	Other	1-3 people	Yes	Daily
327	male	18	Primary	Single	Muslim	RM2,000	Self-employed	Bungalow	4-6 people	Yes	Three times a week
328	Female	35	Primary	Married	Muslim	RM2,000	Civil servant	Other	4-6 people	Yes	Three times a week
329	male	65	Secondary/Certificate	Married	Muslim	RM900	Retiree	Other	1-3 people	No	not cooking
330	male	50	Primary	Married	Muslim	RM900	Self-employed	Other	4-6 people	Yes	Daily

331	male	25	Secondary/Certificate	Married	Muslim	RM900	Self-employed	Other	>6	Yes	Daily
332	male	30	Secondary/Certificate	Married	Muslim	RM4,000	Self-employed	Bungalow	4-6 people	Yes	Three times a week
333	male	25	Diploma/Degree	Single	Muslim	RM900	Student	Other	7	Yes	Daily
334	male	50	Secondary/Certificate	Married	Muslim	RM1,000	Self-employed	Other	4-6 people	Yes	Three times a week
335	Female	50	Primary	Married	Muslim	RM1,000	Self-employed	Other	1-3 people	Yes	Daily
336	Female	25	Diploma/Degree	Married	Muslim	RM4,000	Private-sector employed	Other	1-3 people	Yes	Three times a week
337	male	35	Diploma/Degree	Married	Muslim	RM4,000	Civil servant	Other	1-3 people	Yes	Daily
338	Female	65	Secondary/Certificate	Married	Muslim	RM2,000	Civil servant	Other	4-6 people	Yes	Three times a week

METHODS OF HOUSEHOLD WASTE DISPOSAL

**ID**	**Types of waste generated by household**	**Practice of waste segregation**	**Site of household disposal**	**Transportation of waste**
1	Food debris	No	Appropriate site	own self
2	Food debris	No	Appropriate site	own self
3	Bottles and cans	Yes	Appropriate site	own self
4	Food debris	No	Appropriate site	own self
5	Food debris	No	Appropriate site	own self
6	Bottles and cans	Yes	Appropriate site	own self
7	Bottles and cans	Yes	Appropriate site	own self
8	Food debris	Yes	Appropriate site	own self
9	Food debris	No	Appropriate site	own self
10	Plastics	Yes	Appropriate site	own self

11	Food debris	Yes	Appropriate site	own self
12	Food debris	No	Appropriate site	own self
13	Plastics	No	Appropriate site	own self
14	Plastics	No	Appropriate site	own self
15	Bottles and cans	Yes	Appropriate site	own self
16	Bottles and cans	Yes	Appropriate site	own self
17	Food debris	No	Appropriate site	own self
18	Plastics	No	Appropriate site	Children
19	Food debris	Yes	Appropriate site	own self
20	Food debris	No	Appropriate site	own self
21	Food debris	Yes	Appropriate site	Children
22	Plastics	Yes	Appropriate site	own self
23	Plastics	Yes	Appropriate site	own self
24	Food debris	No	Appropriate site	own self
25	Food debris	No	Appropriate site	own self
26	Bottles and cans	Yes	Appropriate site	Children
27	Food debris	Yes	Appropriate site	own self
28	Food debris	Yes	Appropriate site	own self
29	Food debris	No	Appropriate site	own self
30	Food debris	No	Appropriate site	own self
31	Food debris	No	Appropriate site	paid collector
32	Plastics	Yes	Appropriate site	paid collector
33	Food debris	No	Appropriate site	own self
34	Plastics	Yes	Appropriate site	own self
35	Food debris	Yes	Appropriate site	own self
36	Food debris	Yes	Appropriate site	own self
37	Food debris	No	Appropriate site	own self
38	Food debris	Yes	Appropriate site	paid collector
39	Food debris	Yes	Appropriate site	own self
40	Food debris	No	Appropriate site	own self
41	Food debris	Yes	Appropriate site	own self

42	Food debris	Yes	Appropriate site	own self
43	Food debris	No	Appropriate site	own self
44	Food debris	No	Appropriate site	own self
45	Plastics	No	Appropriate site	own self
46	Plastics	Yes	Appropriate site	own self
47	Food debris	Yes	Appropriate site	own self
48	Food debris	Yes	Appropriate site	own self
49	Food debris	Yes	Appropriate site	own self
50	Food debris	Yes	Appropriate site	Children
51	Food debris	Yes	Appropriate site	own self
52	Food debris	Yes	Appropriate site	own self
53	Food debris	Yes	Appropriate site	Children
54	Food debris	Yes	Appropriate site	own self
55	Food debris	Yes	Appropriate site	own self
56	Food debris	No	Appropriate site	own self
57	Food debris	Yes	Appropriate site	own self
58	Food debris	Yes	Appropriate site	own self
59	Food debris	Yes	Appropriate site	Children
60	Plastics	Yes	Appropriate site	own self
61	Plastics	No	Appropriate site	others
62	Food debris	No	Appropriate site	own self
63	Food debris	Yes	Appropriate site	own self
64	Plastics	No	Appropriate site	paid collector
65	Plastics	Yes	Appropriate site	own self
66	Food debris	No	Appropriate site	Children
67	Food debris	No	Appropriate site	own self
68	Food debris	No	Appropriate site	own self
69	Food debris	No	Appropriate site	own self
70	Plastics	No	Appropriate site	own self
71	Food debris	No	Appropriate site	own self
72	Plastics	Yes	Appropriate site	own self

73	Food debris	Yes	Appropriate site	own self
74	Food debris	Yes	Appropriate site	own self
75	Plastics	Yes	Appropriate site	paid collector
76	Food debris	No	Appropriate site	paid collector
77	Food debris	No	Appropriate site	paid collector
78	Food debris	Yes	Appropriate site	own self
79	Food debris	No	Appropriate site	paid collector
80	Food debris	No	Appropriate site	others
81	Plastics	No	Appropriate site	own self
82	Bottles and cans	No	Appropriate site	own self
83	Food debris	Yes	Appropriate site	own self
84	Food debris	No	Appropriate site	own self
85	Food debris	Yes	Appropriate site	paid collector
86	Plastics	No	Appropriate site	others
87	Food debris	No	Appropriate site	own self
88	Food debris	Yes	Appropriate site	own self
89	Food debris	Yes	Appropriate site	own self
90	Food debris	No	Appropriate site	own self
91	Plastics	No	Appropriate site	others
92	Plastics	No	Appropriate site	own self
93	Plastics	No	Appropriate site	others
94	Food debris	No	Appropriate site	own self
95	Food debris	No	Appropriate site	Children
96	Food debris	Yes	Appropriate site	Children
97	Food debris	No	Appropriate site	own self
98	others	No	Appropriate site	others
99	Food debris	No	Appropriate site	own self
100	Food debris	No	Appropriate site	own self
101	Food debris	No	Appropriate site	own self
102	others	No	Inappropriate	Children
103	Food debris	No	Inappropriate	own self

104	Food debris	Yes	Appropriate site	own self
105	Bottles and cans	Yes	Appropriate site	own self
106	Food debris	No	Appropriate site	own self
107	Food debris	No	Appropriate site	own self
108	Plastics	No	Appropriate site	Children
109	Food debris	Yes	Appropriate site	own self
110	Food debris	No	Inappropriate	own self
111	Food debris	Yes	Appropriate site	own self
112	Food debris	No	Appropriate site	own self
113	Food debris	No	Appropriate site	Children
114	Food debris	No	Appropriate site	own self
115	Food debris	No	Appropriate site	own self
116	Food debris	No	Appropriate site	own self
117	Food debris	Yes	Appropriate site	own self
118	Food debris	Yes	Appropriate site	own self
119	Food debris	No	Appropriate site	own self
120	Food debris	No	Appropriate site	own self
121	Food debris	No	Appropriate site	own self
122	Bottles and cans	Yes	Appropriate site	own self
123	Food debris	No	Appropriate site	own self
124	Plastics	Yes	Appropriate site	own self
125	Food debris	Yes	Appropriate site	own self
126	Food debris	Yes	Appropriate site	Paid collector
127	Food debris	Yes	Appropriate site	own self
128	Food debris	Yes	Appropriate site	own self
129	Food debris	Yes	Appropriate site	own self
130	Food debris	Yes	Appropriate site	own self
131	Food debris	Yes	Appropriate site	own self
132	Food debris	Yes	Appropriate site	Paid collector
133	Food debris	Yes	Appropriate site	own self
134	Plastics	no	Appropriate site	others

135	Food debris	no	Appropriate site	own self
136	Food debris	Yes	Appropriate site	own self
137	Food debris	No	Appropriate site	Paid collector
138	Food debris	Yes	Appropriate site	others
139	Food debris	No	Appropriate site	others
140	Food debris	Yes	Appropriate site	others
141	Food debris	No	Appropriate site	others
142	Food debris	No	Appropriate site	others
143	Food debris	No	Appropriate site	others
144	Plastics	No	Appropriate site	others
145	Food debris	No	Appropriate site	others
146	Food debris	No	Appropriate site	others
147	Food debris	No	Appropriate site	others
148	Food debris	No	Appropriate site	own self
149	Food debris	No	Appropriate site	own self
150	Food debris	No	Appropriate site	own self
151	Food debris	No	Appropriate site	others
152	Food debris	No	Appropriate site	others
153	Food debris	No	Appropriate site	own self
154	Food debris	Yes	Appropriate site	Paid collector
155	Food debris	No	Appropriate site	Paid collector
156	Food debris	No	Appropriate site	own self
157	Plastics	Yes	Appropriate site	own self
158	Food debris	Yes	Appropriate site	own self
159	Food debris	No	Appropriate site	others
160	Plastics	No	Appropriate site	others
161	Plastics	No	Appropriate site	own self
162	Food debris	No	Appropriate site	own self
163	Bottles and cans	Yes	Appropriate site	others
164	Food debris	No	Appropriate site	others
165	Bottles and cans	No	Appropriate site	own self

166	Food debris	No	Appropriate site	own self
167	Plastics	Yes	Appropriate site	own self
168	Food debris	No	Appropriate site	Paid collector
169	Bottles and cans	Yes	Appropriate site	Paid collector
170	Food debris	No	Appropriate site	Paid collector
171	Food debris	Yes	Appropriate site	own self
172	Food debris	Yes	Appropriate site	own self
173	Food debris	Yes	Appropriate site	Paid collector
174	Food debris	Yes	Appropriate site	own self
175	Food debris	Yes	Appropriate site	own self
176	Plastics	No	Appropriate site	Paid collector
177	Plastics	Yes	Appropriate site	Paid collector
178	Food debris	Yes	Appropriate site	own self
179	Food debris	No	Appropriate site	own self
180	Food debris	No	Appropriate site	own self
181	Food debris	No	Appropriate site	own self
182	Plastics	No	Inappropriate	Children
183	Food debris	Yes	Appropriate site	own self
184	Food debris	No	Appropriate site	own self
185	Plastics	No	Appropriate site	own self
186	others	No	Appropriate site	Paid collector
187	Bottles and cans	No	Appropriate site	own self
188	Food debris	Yes	Appropriate site	own self
189	Food debris	Yes	Appropriate site	own self
190	Plastics	Yes	Inappropriate	own self
191	Plastics	Yes	Appropriate site	Paid collector
192	Plastics	Yes	Appropriate site	Paid collector
193	Plastics	Yes	Appropriate site	own self
194	Plastics	Yes	Inappropriate	own self
195	others	No	Appropriate site	own self
196	Food debris	No	Appropriate site	Paid collector

197	Plastics	No	Inappropriate	own self
198	Food debris	Yes	Appropriate site	own self
199	Plastics	Yes	Inappropriate	own self
200	Food debris	No	Appropriate site	own self
201	Plastics	No	Appropriate site	own self
202	Food debris	No	Appropriate site	own self
203	Food debris	Yes	Inappropriate	own self
204	Plastics	No	Appropriate site	own self
205	others	No	Appropriate site	own self
206	Bottles and cans	Yes	Appropriate	Paid collector
207	Plastics	Yes	Appropriate site	Paid collector
208	Plastics	Yes	Appropriate site	own self
209	Plastics	Yes	Appropriate site	own self
210	Food debris	No	Appropriate site	Paid collector
211	Plastics	No	Inappropriate	own self
212	Plastics	Yes	Appropriate site	Paid collector
213	Plastics	Yes	Appropriate site	Paid collector
214	Food debris	No	Appropriate site	own self
215	Food debris	Yes	Appropriate site	own self
216	Food debris	No	Appropriate site	own self
217	food debris	Yes	appropriate site	own self
218	food debris	Yes	Appropriate site	own self
219	Food debris	No	Appropriate site	Children
220	Food debris	Yes	Appropriate site	own self
221	Food debris	No	Appropriate site	own self
222	Food debris	No	Appropriate site	own self
223	Food debris	No	Appropriate site	own self
224	Food debris	No	Appropriate site	own self
225	bottle and tin	No	Inappropriate site	own self
226	Plastics	Yes	Appropriate site	own self
227	Food debris	Yes	Appropriate site	own self

228	Food debris	No	Appropriate site	own self
229	Plastics	No	Inappropriate site	own self
230	Food debris	Yes	Appropriate site	own self
231	Food debris	No	Appropriate site	own self
232	Food debris	No	Appropriate site	own self
233	Food debris	Yes	Appropriate site	own self
234	Food debris	No	Appropriate site	own self
235	Food debris	No	Appropriate site	own self
236	Food debris	No	Appropriate site	own self
237	bottle and tin	No	Appropriate site	own self
238	Food debris	Yes	Appropriate site	others
239	Food debris	No	Appropriate site	paid collector
240	Food debris	No	Appropriate site	own self
241	Food debris	Yes	Appropriate site	others
242	Food debris	Yes	Appropriate site	others
243	Food debris	Yes	Appropriate site	others
244	Food debris	Yes	Appropriate site	others
245	Food debris	No	Appropriate site	own self
246	Food debris	No	Appropriate site	own self
247	Plastics	No	Inappropriate site	own self
248	Food debris	Yes	Appropriate site	others
249	Food debris	Yes	Appropriate site	own self
250	bottle and tin	Yes	Appropriate site	own self
251	Food debris	No	Appropriate site	own self
252	Food debris	No	Appropriate site	own self
253	Plastics	No	Inappropriate site	others
254	Food debris	Yes	Appropriate site	own self
255	Food debris	Yes	Appropriate site	own self
256	Food debris	Yes	Appropriate site	own self
257	Food debris	No	Appropriate site	own self
258	Food debris	No	Appropriate site	own self

259	Food debris	No	Appropriate site	own self
260	Food debris	No	Appropriate site	own self
261	Food debris	No	Appropriate site	own self
262	Plastics	No	Appropriate site	own self
263	Food debris	Yes	Appropriate site	others
264	Food debris	No	Appropriate site	own self
265	Plastics	No	Appropriate site	own self
266	Plastics	No	Appropriate site	own self
267	Food debris	No	Appropriate site	own self
268	Food debris	No	Appropriate site	own self
269	Food debris	No	Appropriate site	paid collector
270	Food debris	Yes	Appropriate site	own self
271	Food debris	No	Appropriate site	own self
272	Food debris	No	Appropriate site	own self
273	Food debris	Yes	Appropriate site	paid collector
274	Food debris	Yes	Appropriate site	own self
275	Food debris	Yes	Appropriate site	own self
276	bottle and tin	Yes	appropriate site	own self
277	Food debris	Yes	Appropriate site	others
278	Food debris	Yes	Appropriate site	others
279	Food debris	Yes	Appropriate site	own self
280	Food debris	Yes	Appropriate site	own self
281	Food debris	Yes	Appropriate site	own self
282	Food debris	Yes	Appropriate site	own self
283	Food debris	Yes	Appropriate site	own self
284	Food debris	Yes	Appropriate site	own self
285	Food debris	Yes	Appropriate site	own self
286	Food debris	Yes	Appropriate site	own self
287	Food debris	Yes	Appropriate site	own self
288	bottle and tin	Yes	appropriate site	own self
289	Food debris	Yes	Appropriate site	own self

290	Food debris	Yes	Appropriate site	own self
291	Food debris	Yes	Appropriate site	own self
292	Food debris	Yes	Appropriate site	own self
293	Food debris	Yes	Appropriate site	own self
294	Plastics	Yes	Appropriate site	own self
295	Plastics	Yes	Appropriate site	own self
296	Food debris	Yes	Appropriate site	own self
297	Food debris	Yes	Appropriate site	own self
298	Food debris	Yes	Appropriate site	own self
299	Food debris	Yes	Appropriate site	own self
300	Food debris	Yes	Appropriate site	own self
301	Food debris	Yes	Appropriate site	own self
302	Plastics	Yes	Appropriate site	own self
303	Food debris	Yes	Appropriate site	others
304	Food debris	Yes	Appropriate site	own self
305	Food debris	Yes	Appropriate site	own self
306	Plastics	Yes	Appropriate site	own self
307	Food debris	Yes	Appropriate site	own self
308	Food debris	Yes	Inappropriate site	own self
309	Food debris	Yes	Appropriate site	own self
310	Food debris	No	Appropriate site	own self
311	Food debris	No	Appropriate site	own self
312	Food debris	No	Appropriate site	own self
313	Food debris	Yes	Appropriate site	own self
314	Food debris	Yes	Appropriate site	own self
315	Plastics	Yes	Appropriate site	own self
316	Food debris	No	Appropriate site	own self
317	Food debris	No	Appropriate site	Children
318	Food debris	Yes	Appropriate site	own self
319	Food debris	No	Appropriate site	own self
320	Plastics	No	Appropriate site	Children

321	Food debris	Yes	Appropriate site	own self
322	Food debris	No	Appropriate site	own self
323	Food debris	Yes	Appropriate site	own self
324	Food debris	No	Appropriate site	own self
325	Food debris	No	Appropriate site	own self
326	Food debris	No	Appropriate site	own self
327	Food debris	No	Appropriate site	own self
328	Food debris	Yes	Appropriate site	own self
329	Food debris	Yes	Appropriate site	own self
330	Food debris	Yes	Appropriate site	own self
331	Food debris	Yes	Appropriate site	own self
332	Food debris	Yes	Appropriate site	own self
333	Food debris	No	Appropriate site	own self
334	Plastics	No	Appropriate site	Children
335	Food debris	No	Appropriate site	own self
336	Food debris	No	Appropriate site	own self
337	Food debris	Yes	Appropriate site	own self
338	Food debris	No	Appropriate site	own self

PERCEPTION OF HOUSEHOLD TOWARD WASTE MANAGEMENT

ID	Importance of waste management	Responsible party to clean the residential area	Improper waste management contribute to disease occurrence	The disease caused by mismanagement of waste disposal	The household members are educated on proper waste disposal	Element that motivate the household in waste disposal
1	It is important	residence	yes	Diarrhea	Yes	Cleanliness
2	It is important	residence	yes	Others	Yes	Fear of illness
3	It is important	residence	yes	Diarrhea	Yes	Cleanliness
4	It is important	residence	yes	Others	No	Cleanliness
5	It is important	residence	yes	Malaria	No	Cleanliness

6	It is important	residence	yes	Others	Yes	Cleanliness
7	It is important	residence	yes	Malaria	Yes	Cleanliness
8	It is important	residence	yes	Others	Yes	Cleanliness
9	It is important	residence	yes	Others	Yes	Fear of illness
10	It is important	residence	yes	Diarrhea	Yes	Cleanliness
11	It is important	residence	yes	Others	Yes	Cleanliness
12	It is important	residence	yes	Others	Yes	Odour
13	It is important	residence	yes	Diarrhea	Yes	Fear of illness
14	It is important	residence	yes	Others	Yes	Cleanliness
15	It is important	residence	yes	Others	Yes	Cleanliness
16	It is important	residence	yes	Others	Yes	Cleanliness
17	It is important	residence	yes	Others	Yes	Cleanliness
18	It is important	residence	yes	Typhoid	Yes	Cleanliness
19	It is important	residence	yes	Others	Yes	Cleanliness
20	It is important	residence	yes	Others	Yes	Cleanliness
21	It is important	residence	yes	Others	Yes	Cleanliness
22	It is important	residence	yes	Typhoid	Yes	Cleanliness
23	It is important	residence	yes	Typhoid	Yes	Cleanliness
24	It is important	residence	yes	Diarrhea	Yes	Cleanliness
25	It is important	residence	yes	Malaria	Yes	Cleanliness
26	It is important	residence	yes	Typhoid	Yes	Cleanliness
27	It is important	residence	yes	Typhoid	Yes	Cleanliness
28	It is important	residence	yes	Others	Yes	Cleanliness
29	It is important	residence	yes	Others	No	Cleanliness
30	It is important		no	Others	Yes	Cleanliness
31	It is important	community	yes	Malaria	No	Cleanliness
32	It is important	residence	yes	Others	Yes	Cleanliness
33	It is important	residence	yes	Diarrhea	Yes	Cleanliness
34	It is important	district council	yes	Others	Yes	Cleanliness
35	It is important	residence	yes	Diarrhea	Yes	Cleanliness
36	It is important	residence	yes	Diarrhea	Yes	Cleanliness

37	It is important	residence	yes	Malaria	Yes	Cleanliness
38	It is important	residence	yes	Others	Yes	Cleanliness
39	It is important	residence	yes	Others	No	Odour
40	It is important	residence	yes	Malaria	Yes	Odour
41	It is important	residence	yes	Others	Yes	Cleanliness
42	It is important	district council	yes	Others	Yes	Cleanliness
43	It is important	residence	yes	Others	Yes	Fear of illness
44	It is important	residence	yes	Others	Yes	Cleanliness
45	It is important	residence	yes	Others	Yes	Cleanliness
46	It is important	residence	yes	Others	Yes	Cleanliness
47	It is important	residence	yes	Malaria	No	Cleanliness
48	It is important	residence	no	Malaria	No	Odour
49	It is important	residence	yes	Malaria	Yes	Cleanliness
50	It is important	residence	yes	Others	Yes	Cleanliness
51	It is important	residence	yes	Others	Yes	Odour
52	It is important	residence	yes	Diarrhea	Yes	Fear of illness
53	It is important	residence	yes	Diarrhea	Yes	Odour
54	It is important	residence	yes	Malaria	No	Cleanliness
55	It is important	residence	yes	Malaria	Yes	Cleanliness
56	It is important	residence	yes	Malaria	No	Cleanliness
57	It is important	residence	yes	Malaria	Yes	Fear of illness
58	It is important	residence	yes	Diarrhea	Yes	Cleanliness
59	It is important	residence	yes	Diarrhea	Yes	Cleanliness
60	It is important	residence	yes	Others	Yes	Cleanliness
61	It is important	residence	yes	Diarrhea	Yes	Odour
62	It is important	community	yes	Malaria	Yes	Cleanliness
63	It is important	district council	yes	Others	Yes	Cleanliness
64	It is important	district council	yes	Malaria	No	Fear of illness
65	It is important	district council	yes	Malaria	Yes	Cleanliness
66	It is important	residence	yes	Others	No	Cleanliness
67	It is important	residence	yes	Others	Yes	Cleanliness

68	It is important	district council	yes	Malaria	Yes	Cleanliness
69	It is important	residence	yes	Others	Yes	Fear of illness
70	It is important	residence	yes	Others	Yes	Cleanliness
71	It is important	district council	yes	Diarrhea	Yes	Cleanliness
72	It is important	residence	yes	Others	Yes	Cleanliness
73	It is important	residence	yes	Malaria	Yes	Fear of illness
74	It is important	district council	yes	Typhoid	Yes	Cleanliness
75	It is important	residence	yes	Others	Yes	Cleanliness
76	It is important	residence	yes	Others	Yes	Cleanliness
77	It is important	residence	yes	Malaria	Yes	Cleanliness
78	It is important	residence	yes	Malaria	Yes	Cleanliness
79	It is important	residence	yes	Others	Yes	Cleanliness
80	It is important	district council	no	Others	Yes	Cleanliness
81	It is important	residence	yes	Others	Yes	Cleanliness
82	It is important	district council	yes	Diarrhea	Yes	Cleanliness
83	It is important	residence	yes	Diarrhea	Yes	Cleanliness
84	It is important	community	yes	Diarrhea	Yes	Cleanliness
85	It is important	residence	yes	Others	Yes	Cleanliness
86	It is important	district council	yes	Diarrhea	Yes	Cleanliness
87	It is important	district council	yes	Diarrhea	Yes	Fear of illness
88	It is important	residence	yes	Diarrhea	Yes	Cleanliness
89	It is important	community	yes	Malaria	Yes	Cleanliness
90	It is important	residence	yes	Diarrhea	Yes	Cleanliness
91	It is important	residence	yes	Diarrhea	Yes	Cleanliness
92	It is important	residence	yes	Others	Yes	Odour
93	It is important	residence	yes	Malaria	Yes	Cleanliness
94	It is important	residence	no	Diarrhea	Yes	Odour
95	It is important	residence	yes	Others	No	Cleanliness
96	It is important	residence	yes	Others	Yes	Cleanliness
97	It is important	residence	yes	Others	Yes	Cleanliness
98	It is important	residence	yes	Others	No	Odour

99	It is important	residence	yes	Others	Yes	Cleanliness
100	It is important	residence	yes	Others	No	Cleanliness
101	It is important	residence	yes	Others	Yes	Cleanliness
102	It is important	residence	yes	Others	Yes	Cleanliness
103	It is important	residence	yes	Others	Yes	Odour
104	It is important	residence	yes	Others	Yes	Cleanliness
105	It is important	residence	yes	Others	Yes	Cleanliness
106	It is important	residence	yes	Others	Yes	Cleanliness
107	It is important	residence	yes	Others	Yes	Cleanliness
108	It is important	residence	yes	Typhoid	Yes	Cleanliness
109	It is important	residence	yes	Others	Yes	Cleanliness
110	It is important	residence	yes	Others	Yes	Cleanliness
111	It is important	private waste operator	not sure	Others	Yes	Cleanliness
112	it is not important	district council	yes	Diarrhea	No	Cleanliness
113	It is important	residence	yes	Diarrhea	Yes	Cleanliness
114	It is important	district council	yes	Diarrhea	Yes	Cleanliness
115	It is important	residence	yes	Malaria	Yes	Cleanliness
116	It is important	residence	yes	Others	Yes	Fear of sickness
117	It is important	residence	yes	Typhoid	Yes	Cleanliness
118	It is important	residence	yes	Others	Yes	Cleanliness
119	It is important	residence	yes	Others	No	Cleanliness
120	It is important	district council	yes	Others	Yes	Cleanliness
121	It is important	residence	yes	diarrhea	Yes	Cleanliness
122	It is important	district council	yes	diarrhea	Yes	Odour
123	It is important	residence	yes	diarrhea	Yes	Cleanliness
124	It is important	residence	yes	Others	Yes	Fear of sickness
125	It is important	residence	yes	Others	Yes	Cleanliness
126	It is important	district council	yes	Driarrhea	Yes	Cleanliness
127	It is important	district council	yes	Malaria	Yes	Cleanliness

128	It is important	private waste operator	yes	Diarrhea	Yes	Cleanliness
129	It is important	private waste operator	yes	Others	Yes	Fear of sickness
130	It is important	district council	yes	Malaria	Yes	Cleanliness
131	It is important	district council	yes	diarrhea	No	Cleanliness
132	It is important	district council	yes	diarrhea	Yes	Cleanliness
133	It is important	private waste operator	yes	Typhoid	Yes	Cleanliness
134	It is important	residence	yes	Others	Yes	Cleanliness
135	It is important	district council	yes	Malaria	Yes	Cleanliness
136	It is important	private waste operator	yes	Others	Yes	Cleanliness
137	It is important	district council	yes	Typhoid	Yes	Cleanliness
138	It is important	residence	yes	Others	Yes	Cleanliness
139	It is important	district council	yes	Diarrhea	Yes	Fear of sickness
140	It is important	district council	yes	Others	Yes	Odour
141	It is important	residence	yes	Others	Yes	Cleanliness
142	It is important	residence	yes	Others	Yes	Cleanliness
143	It is important	district council	yes	Others	Yes	Cleanliness
144	It is important	residence	yes	Others	Yes	Cleanliness
145	It is important	residence	yes	Others	Yes	Cleanliness
146	It is important	residence	yes	Others	Yes	Cleanliness
147	It is important	residence	yes	Others	Yes	Cleanliness
148	It is important	residence	yes	Diarrhea	Yes	Fear of sickness
149	It is important	residence	yes	Others	Yes	Cleanliness
150	It is important	district council	yes	Diarrhea	Yes	Fear of sickness
151	It is important	residence	yes	Diarrhea	Yes	Cleanliness
152	It is important	residence	yes	Others	Yes	Cleanliness
153	It is important	district council	yes	Others	Yes	Cleanliness
154	It is important	district council	yes	Diarrhea	Yes	Cleanliness
155	It is important	district council	yes	Malaria	Yes	Cleanliness

156	It is important	residence	not sure	Others	Yes	Cleanliness
157	It is important	district council	yes	Malaria	Yes	Fear of sickness
158	It is important	district council	no	Diarrhea	Yes	Odour
159	It is important	residence	yes	Others	Yes	Cleanliness
160	It is important	residence	yes	Diarrhea	Yes	Cleanliness
161	It is important	residence	yes	Others	Yes	Cleanliness
162	It is important	residence	yes	Others	Yes	Cleanliness
163	It is important	district council	yes	Others	Yes	Cleanliness
164	It is important	residence	yes	Malaria	Yes	Cleanliness
165	It is important	district council	yes	Diarrhea	Yes	Cleanliness
166	It is important	district council	yes	Others	Yes	Cleanliness
167	It is important	district council	yes	Malaria	Yes	Cleanliness
168	It is important	residence	yes	Others	Yes	Cleanliness
169	It is important	district council	yes	Others	Yes	Cleanliness
170	It is important	residence	yes	Diarrhea	Yes	Cleanliness
171	It is important	district council	yes	Diarrhea	Yes	Odour
172	It is important	district council	yes	malaria	Yes	Cleanliness
173	It is important	residence	not sure	Others	Yes	Odour
174	It is important	district council	yes	Malaria	Yes	Cleanliness
175	It is important	district council	yes	Typhoid	Yes	Cleanliness
176	It is important	district council	yes	Others	Yes	Cleanliness
177	It is important	residence	yes	Others	Yes	Cleanliness
178	It is important	district council	yes	Others	Yes	Cleanliness
179	It is important	residence	yes	Others	Yes	Cleanliness
180	It is important	residence	yes	Others	Yes	Cleanliness
181	It is important	residence	yes	Typhoid	Yes	Cleanliness
182	It is important	residence	not sure	Diarrhea	Yes	Fear of sickness
183	It is important	residence	yes	Others	Yes	Cleanliness
184	It is important	district council	yes	Diarrhea	Yes	Fear of sickness
185	It is important	residence	yes	Malaria	Yes	Odour
186	It is important	district council	yes	Diarrhea	Yes	Cleanliness

187	It is important	residence	yes	Malaria	Yes	Fear of sickness
188	It is important	district council	yes	Others	Yes	Fear of sickness
189	It is important	residence	yes	Others	Yes	Cleanliness
190	It is important	residence	yes	Others	Yes	Cleanliness
191	It is important	residence	yes	Others	Yes	Cleanliness
192	It is important	district council	yes	Others	Yes	Cleanliness
193	It is important	residence	yes	Malaria	Yes	cleanliness
194	It is important	residence	yes	Others	Yes	Cleanliness
195	It is important	residence	yes	Diarrhea	Yes	Cleanliness
196	It is important	district council	yes	Others	Yes	Cleanliness
197	It is important	residence	yes	Others	Yes	Odour
198	It is important	residence	yes	Malaria	Yes	Cleanliness
199	It is important	residence	yes	Others	Yes	Cleanliness
200	It is important	residence	yes	Others	Yes	Cleanliness
201	It is important	residence	yes	Others	Yes	Cleanliness
202	It is important	residence	yes	Malaria	Yes	Cleanliness
203	It is important	residence	yes	Malaria	Yes	Fear of sickness
204	It is important	residence	yes	Others	Yes	Cleanliness
205	It is important	residence	yes	Malaria	Yes	Fear of sickness
206	It is important	residence	yes	Others	Yes	Cleanliness
207	It is important	district council	yes	Others	Yes	Cleanliness
208	It is important	residence	yes	Typhoid	Yes	cleanliness
209	It is important	residence	yes	Others	Yes	Cleanliness
210	It is important	residence	yes	Malaria	Yes	Cleanliness
211	It is important	residence	yes	Others	Yes	Cleanliness
212	It is important	district council	yes	Others	Yes	Fear of sickness
213	It is important	residence	yes	Malaria	Yes	Cleanliness
214	It si important		yes	Diarrhea	Yes	Cleanliness
215	It is important	residence	yes	Diarrhea	Yes	Cleanliness
216	It is important	residence	yes	Others	Yes	Cleanliness
217	it is important	residence	yes	typhoid	Yes	Cleanliness

218	it is important	community	yes	Diarrhea	Yes	Cleanliness
219	It is important	community	yes	Diarrhea	Yes	Cleanliness
220	It is important	community	yes	Diarrhea	Yes	Fear of illness
221	It is important	community	yes	Diarrhea	Yes	Fear of illness
222	It is important	district council	yes	Diarrhea	Yes	Fear of illness
223	It is important	district council	yes	Diarrhea	Yes	Fear of illness
224	It is important	district council	yes	Diarrhea	Yes	Cleanliness
225	It is important	residence	yes	Others	Yes	Cleanliness
226	It is important	community	yes	Diarrhea	Yes	Cleanliness
227	It is important	residence	yes	Others	Yes	Cleanliness
228	It is important	residence	yes	Diarrhea	Yes	Fear of illness
229	It is important	community	yes	Others	Yes	Cleanliness
230	It is important	community	yes	Diarrhea	Yes	Cleanliness
231	It is important	community	yes	malaria	Yes	Fear of illness
232	It is important	district council	yes	Diarrhea	Yes	Cleanliness
233	It is important	community	yes	Others	Yes	Cleanliness
234	It is important	community	yes	Diarrhea	Yes	Fear of illness
235	It is important	community	yes	Others	Yes	Cleanliness
236	It is important	community	yes	Diarrhea	Yes	Fear of illness
237	It is important	community	yes	malaria	Yes	Fear of illness
238	It is important	district council	yes	typhoid	Yes	Cleanliness
239	It is important	residence	yes	malaria	Yes	Cleanliness
240	It is important	district council	yes	Diarrhea	Yes	Cleanliness
241	It is important	district council	yes	Diarrhea	Yes	Cleanliness
242	It is important	residence	yes	Diarrhea	Yes	Cleanliness
243	It is important	community	yes	Diarrhea	Yes	Cleanliness
244	It is important	residence	yes	malaria	Yes	Cleanliness
245	It is important	community	yes	Diarrhea	Yes	Cleanliness
246	It is important	district council	yes	Others	Yes	Cleanliness
247	It is important	district council	yes	Diarrhea	Yes	Cleanliness
248	It is important	community	yes	Diarrhea	Yes	Cleanliness

249	It is important	community	yes	Diarrhea	Yes	Cleanliness
250	It is important	community	yes	Diarrhea	Yes	Cleanliness
251	It is important	community	yes	Diarrhea	Yes	Fear of illness
252	It is important	residence	yes		Yes	Fear of illness
253	It is important	community	yes	Others	Yes	Cleanliness
254	It is important	district council	yes	malaria	Yes	Cleanliness
255	It is important	district council	yes	malaria	Yes	Cleanliness
256	It is important	residence	yes	Diarrhea	Yes	Cleanliness
257	It is important	district council	yes	Others	Yes	Cleanliness
258	It is important	community	yes	malaria	Yes	Cleanliness
259	It is important	residence	yes	Diarrhea	Yes	odor
260	It is important	community	yes	Others	Yes	Cleanliness
261	It is important	residence	yes	malaria	Yes	Cleanliness
262	It is important	community	yes	malaria	Yes	Cleanliness
263	It is important	district council	yes	Diarrhea	Yes	Cleanliness
264	It is important	community	yes	malaria	Yes	Cleanliness
265	It is important	community	yes	malaria	Yes	Cleanliness
266	It is important	residence	yes	Diarrhea	Yes	Cleanliness
267	It is important	district council	yes	Diarrhea	Yes	Cleanliness
268	It is important	residence	yes	Others	Yes	Cleanliness
269	It is important	district council	yes	typhoid	Yes	Cleanliness
270	It is important	community	yes	Others	Yes	Cleanliness
271	It is important	residence	yes	Diarrhea	Yes	Cleanliness
272	It is important	residence	yes	Diarrhea	Yes	Cleanliness
273	It is important	residence	yes	Diarrhea	Yes	Cleanliness
274	It is important	residence	yes	malaria	Yes	Cleanliness
275	It is important	community	yes	Diarrhea	Yes	Cleanliness
276	It is important	district council	yes	Diarrhea	Yes	Cleanliness
277	It is important	district council	yes	Diarrhea	Yes	Cleanliness
278	It is important	district council	yes	malaria	Yes	Cleanliness
279	It is important	community	yes	Diarrhea	Yes	Cleanliness

280	It is important	community	yes	Diarrhea	Yes	Fear of illness
281	It is important	district council	yes	malaria	Yes	Cleanliness
282	It is important	community	yes	Diarrhea	Yes	Cleanliness
283	It is important	community	yes	Diarrhea	Yes	Cleanliness
284	It is important	residence	yes	malaria	Yes	Cleanliness
285	It is important	residence	yes	malaria	Yes	Cleanliness
286	It is important	residence	yes	malaria	Yes	Cleanliness
287	It is important	residence	yes	malaria	Yes	Cleanliness
288	It is important	residence	yes	malaria	Yes	Cleanliness
289	It is important	district council	yes	Others	Yes	Cleanliness
290	It is important	community	yes	typhoid	Yes	Cleanliness
291	It is important	community	yes	malaria	Yes	Cleanliness
292	It is important	district council	yes	Diarrhea	Yes	Cleanliness
293	It is important	district council	yes	Diarrhea	Yes	Cleanliness
294	It is important	district council	yes	Diarrhea	Yes	odor
295	It is important	district council	yes	Diarrhea	Yes	Fear of illness
296	It is important	district council	yes	Diarrhea	Yes	Fear of illness
297	It is important	community	yes	malaria	Yes	Cleanliness
298	It is important	district council	yes	Diarrhea	Yes	Fear of illness
299	It is important	district council	yes	malaria	Yes	Cleanliness
300	It is important	residence	yes	Others	Yes	Cleanliness
301	It is important	residence	yes	Diarrhea	Yes	Cleanliness
302	It is important	district council	yes	malaria	Yes	Cleanliness
303	It is important	district council	yes	Diarrhea	Yes	odor
304	It is important	district council	yes	Diarrhea	Yes	Cleanliness
305	It is important	district council	yes	Diarrhea	Yes	Cleanliness
306	It is important	residence	yes	Malaria	Yes	Cleanliness
307	It is important	residence	yes	Others	Yes	Cleanliness
308	It is important	residence	yes	Diarrhea	Yes	Cleanliness
309	It is important	residence	yes	Malaria	Yes	Cleanliness
310	It is important	residence	yes	Malaria	Yes	Cleanliness

311	It is important	residence	yes	Diarrhea	Yes	Cleanliness
312	It is important	residence	yes	Malaria	No	Cleanliness
313	It is important	residence	yes	Diarrhea	Yes	Cleanliness
314	It is important	residence	yes	Malaria	Yes	Fear of illness
315	It is important	residence	yes	Others	Yes	Cleanliness
316	It is important	residence	yes	Diarrhea	No	Cleanliness
317	It is important	district council	yes	Malaria	No	Cleanliness
318	It is important	residence	yes	Malaria	Yes	Fear of illness
319	It is important	residence	yes	Diarrhea	No	Cleanliness
320	It is important	residence	no	Others	No	Cleanliness
321	It is important	residence	yes	Others	Yes	Cleanliness
322	It is important	residence	yes	Malaria	Yes	Cleanliness
323	It is important	residence	yes	Typhoid	Yes	Cleanliness
324	It is important	residence	yes	Malaria	Yes	Fear of illness
325	It is important	residence	no	Others	No	Cleanliness
326	It is important	residence	yes	Typhoid	Yes	Cleanliness
327	It is important	residence	yes	Others	Yes	Cleanliness
328	It is important	residence	yes	Others	Yes	Cleanliness
329	It is important	residence	yes	Others	Yes	Cleanliness
330	It is important	residence	yes	Others	Yes	Cleanliness
331	It is important	residence	yes	Typhoid	Yes	Cleanliness
332	It is important	residence	yes	Typhoid	Yes	Cleanliness
333	It is important	residence	yes	Others	No	Cleanliness
334	It is important	residence	not sure	Others	Yes	Cleanliness
335	It is important	residence	no	Others	Yes	Cleanliness
336	It is important	residence	yes	Diarrhea	Yes	Cleanliness
337	It is important	residence	yes	Diarrhea	Yes	Cleanliness
338	It is important	residence	yes	Malaria	Yes	Cleanliness
